# The Logic of EGFR/ErbB Signaling: Theoretical Properties and Analysis of High-Throughput Data

**DOI:** 10.1371/journal.pcbi.1000438

**Published:** 2009-08-07

**Authors:** Regina Samaga, Julio Saez-Rodriguez, Leonidas G. Alexopoulos, Peter K. Sorger, Steffen Klamt

**Affiliations:** 1 Max Planck Institute for Dynamics of Complex Technical Systems, Magdeburg, Germany; 2 Department of Systems Biology, Harvard Medical School, Boston, Massachusetts, United States of America; 3 Department of Biological Engineering, Massachusetts Institute of Technology, Cambridge, Massachusetts, United States of America; 4 Department of Mechanical Engineering, National Technical University of Athens, Athens, Greece; California Institute of Technology, United States of America

## Abstract

The epidermal growth factor receptor (EGFR) signaling pathway is probably the best-studied receptor system in mammalian cells, and it also has become a popular example for employing mathematical modeling to cellular signaling networks. Dynamic models have the highest explanatory and predictive potential; however, the lack of kinetic information restricts current models of EGFR signaling to smaller sub-networks. This work aims to provide a large-scale qualitative model that comprises the main and also the side routes of EGFR/ErbB signaling and that still enables one to derive important functional properties and predictions. Using a recently introduced logical modeling framework, we first examined general topological properties and the qualitative stimulus-response behavior of the network. With species equivalence classes, we introduce a new technique for logical networks that reveals sets of nodes strongly coupled in their behavior. We also analyzed a model variant which explicitly accounts for uncertainties regarding the logical combination of signals in the model. The predictive power of this model is still high, indicating highly redundant sub-structures in the network. Finally, one key advance of this work is the introduction of new techniques for assessing high-throughput data with logical models (and their underlying interaction graph). By employing these techniques for phospho-proteomic data from primary hepatocytes and the HepG2 cell line, we demonstrate that our approach enables one to uncover inconsistencies between experimental results and our current qualitative knowledge and to generate new hypotheses and conclusions. Our results strongly suggest that the Rac/Cdc42 induced p38 and JNK cascades are independent of PI3K in both primary hepatocytes and HepG2. Furthermore, we detected that the activation of JNK in response to neuregulin follows a PI3K-dependent signaling pathway.

## Author Summary

The epidermal growth factor receptor (EGFR) signaling pathway is arguably the best-characterized receptor system in mammalian cells and has become a prime example for mathematical modeling of cellular signal transduction. Most of these models are constructed to describe dynamic and quantitative events but, due to the lack of precise kinetic information, focus only on certain regions of the network. Qualitative modeling approaches relying on the network structure provide a suitable way to deal with large-scale networks as a whole. Here, we constructed a comprehensive qualitative model of the EGFR/ErbB signaling pathway with more than 200 interactions reflecting our current state of knowledge. A theoretical analysis revealed important topological and functional properties of the network such as qualitative stimulus-response behavior and redundant sub-structures. Subsequently, we demonstrate how this qualitative model can be used to assess high-throughput data leading to new biological insights: comparing qualitative predictions (such as expected “ups” and “downs” of activation levels) of our model with experimental data from primary human hepatocytes and from the liver cancer cell line HepG2, we uncovered inconsistencies between measurements and model structure. These discrepancies lead to modifications in the EGFR/ErbB signaling network relevant at least for liver biology.

## Introduction

The epidermal growth factor receptor (EGFR) signaling pathway is among the best studied receptor systems in mammalian cells. Signaling through EGFR (ErbB1) and its family members ErbB2 (Her2/Neu2) ErbB3 and ErbB4 regulates cellular processes such as survival, proliferation, differentiation and motility and ErbB receptors are important targets for new and existing anti-cancer drugs [1],[2].

Mathematical modeling of the EGFR system started more than 25 years ago with efforts to describe binding to and internalization of the receptor [3] that was followed by a variety of dynamic models that deal with different aspects of the system (reviewed in [4],[5]). Whereas the first EGFR models focused on the receptor itself – internalization, ligand binding, and receptor homodimerization [6] – later models included downstream signaling events (e.g. [7]–[9]). More recent studies also address homo- and hetero-dimerization among members of the ErbB receptor family and the effects on downstream of binding to different ligands (of which 13 are known; e.g. [10]–[13]). All these models describe aspects of EGFR/ErbB signaling with a set of stoichiometric reactions and the dynamics of the involved species is described by a set of ordinary differential equations (ODEs). In order to simulate the model, the kinetic constants and initial concentrations of the model have to be known or, more likely, they must be estimated.

Recently, a large-scale map was constructed by Kitano and colleagues to capture the current state of knowledge about interactions in the EGFR system as a stoichiometric network [14]. This model contains no information on the reaction kinetics and is thus static and cannot be used to perform dynamic simulations. Nonetheless, the Kitano map provides a reasonably comprehensive list of molecules and interactions involved in EGF signaling and represents an excellent starting point for studying its global architecture [14]–[16]. Existing ODE-based models cover only limited parts of the map, and parametric uncertainty present even in these smaller models suggests that it is not currently practical to build an ODE model of the entire pathway having high explanatory and predictive power. Instead, structural and qualitative (parameter-free) modeling approaches is the tool of choice. In fact, many important properties of a system rely solely on the often well-known network structure, including many that govern dynamic behavior; feedback loops, for example, are captured in the wiring diagram.

Whereas structural (stoichiometric) analysis of metabolic networks is quite well established [17], relatively few efforts have been made thus far to study qualitatively the propagation of information in signaling networks. Efforts to date include statistical analyses of interaction graphs of large-scale protein-protein networks (e.g. [18]) and other approaches that rely on graph theory (e.g. [15],[19]). Petri net theory [20],[21] and constraint-based modeling [22] have also been used to unravel structural properties of signal transduction networks.

Boolean (discrete logic) description of interaction networks has quite a long tradition in theoretical biology. In the past, it has been mainly applied to random networks [23] or gene regulatory networks of moderate size (e.g. [24]–[27]). However, we have recently developed a Boolean framework that is specifically tailored to signaling networks. In contrast to gene regulatory networks, signaling networks are usually structured into input, processing and output layers. This approach has recently been applied successfully to a large-scale model of T cell signaling [28], and used in concert with high-throughput data to analyze cell-specific network topologies (Saez-Rodriguez *et al*, in preparation).

Within this framework, we have set-up a logical model of the main parts of the stoichiometric model of EGFR signaling [14] and additionally of signaling through ErbB2, ErbB3 and ErbB4. As mentioned above, the stoichiometric model of Oda *et al* [14] does not allow for dynamic simulations. Also functional issues related to network structure can be studied only to a minor extent because the stoichiometric model is limited regarding the analysis of signal flows relevant in signaling networks. By translating the stoichiometric (mass-flow based) into a logical (signal-flow based) representation, we obtain an executable model facilitating functional predictions about input-output responses of a very complex signaling cascade. Our model comprises 104 species and 204 interactions and is among the largest of a mammalian signaling network but we have recently become aware of the interesting work of Helikar *et al* [29] who also studied a large-scale Boolean network containing parts of the EGFR/ErbB induced signaling pathways. Their work focuses on a statistical analysis of the possible (non-deterministic) discrete behaviors of their Boolean model. In contrast, our model provides deterministic and testable predictions about responses and we have verified many using functional data. In the process, we have uncovered non-obvious functional properties of the ErbB signaling pathway that are likely to be biologically significant.

This paper is organized as follows: the first part describes how we translated the stoichiometric EGFR/ErbB model of Oda *et al* [14] into a logical model via a set of general rules. The second part presents results from a theoretical analysis of the network including, for example, a characterization of feedback structure and identification of network components whose behavior is strongly coupled. The final section describes application of the logical model to interpret functional data in which primary human hepatocytes and hepatocarcinoma cell line HepG2 were exposed to different ErbB ligands in combination with inhibitors of intracellular signaling kinases. We show that a Boolean model of ErbB signaling can generate experimentally verifiable predictions about input-output behavior in the face of perturbation and that new hypotheses about biological function can be generated

## Results

### From a stoichiometric to a logical model for EGFR/ErbB signaling

**Figure 1:**
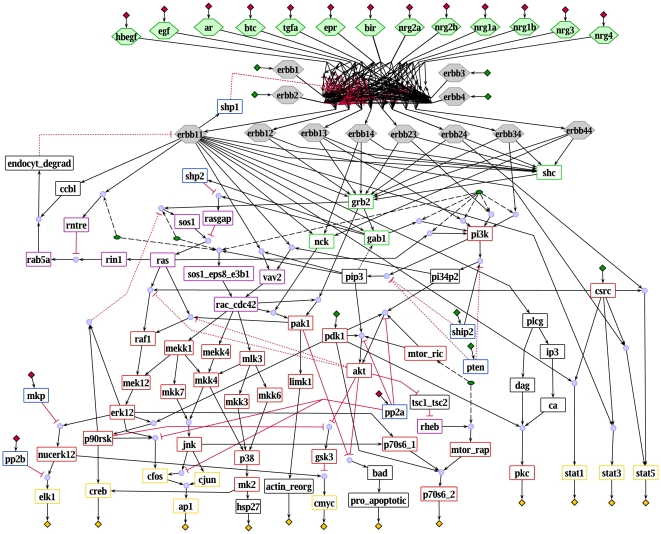
Logical model of the EGF-/ErbB receptor signaling pathway represented in *ProMoT*. Blue circles symbolize AND connections. Inputs with default value 0 are indicated with red diamonds, inputs with default value 1 by green diamonds. Yellow diamonds stand for the outputs of the model. Gray hexagons represent the receptors (homodimers as well as heterodimers) and green hexagons stand for the 13 different ligands. Green ellipses symbolize reservoirs. The remaining species (symbolized with rectangles) are colored according to their function: red: kinases; blue: phosphatases; yellow: transcription factors; green: adaptor molecules; violet: small G proteins as well as GAPs and GEFs; black: other. The box in the upper part of the network contains binding of the ligands to the receptor and receptor dimerization, showing the high combinatorial complexity. Black arrows indicate activations, red blunt-ended lines stand for inhibitions. Dotted lines represent “late” interactions (with attribute τ = 2) that are excluded when studying the initial network response. Dashed lines indicate connections from reservoirs. Dummy species (see Methods) are not displayed.

Based on a stoichiometric model of EGF receptor signaling [14] and additional information from the literature, we built a logical model that describes signaling induced by 13 members of the EGF ligand family through ErbB1-4, leading to the activation of various kinases and transcription factors that effect proliferation, growth and survival (see Figure 1 and Table S1). Ligand binding causes the formation of eight different ErbB-dimers that autophosphorylate and then provide docking sites for adaptor proteins such as Gab1, Grb2 and Shc, which transmit signals to the small G proteins Ras and Rac, leading to the activation of MAPK cascades. Among these, ERK1/2 is the best studied but our model also comprises the JNK and p38 cascades. Highly interconnected with the MAPKs and also downstream of the ErbB receptors is PI3K/Akt signaling, another major branch of the model. Furthermore, activation of different STATs and the PLCγ/PKC pathway are included.

Our model contains most parts of the stoichiometric model of Oda *et al* [14]. However, endocytosis, the G1/S transition of the cell cycle as well as the crosstalk with the G protein coupled receptor signaling cascade are not considered in our model as we focus here on early signaling events induced by external stimuli (EGF-type ligands). In contrast, our model considers signaling through all different ErbB dimers (in addition to EGFR homodimers), which was not part of the stoichiometric model (though a simplified diagram has been given in [14]). Finally, there are some reactions and species that are only contained in the logical model so as to use the data set (e.g. the mammalian target of rapamycin (mTOR), p70S6 kinase). Differences between the stoichiometric and the logical model regarding considered components and interactions are also explained in the model documentation (see Table S1).

**Figure 2:**
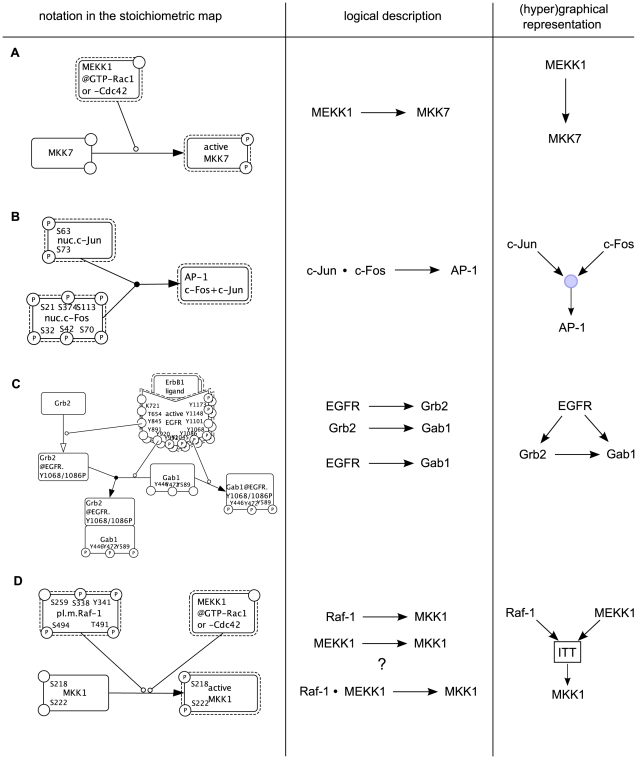
Examples illustrating the translation of the stoichiometric EGFR model into a logical description. The examples are taken from the stoichiometric map of Oda *et al* [14]. **A** The activation level of MKK7 is only influenced by one upstream molecule (active MEKK1). **B** c-Jun and c-Fos form the transcription factor AP-1. Accordingly, both species are combined with an AND gate (denoted by “·” in the logical equations). **C** Gab1 can bind directly to EGFR homodimers or via receptor-bound Grb2. For the activation of downstream elements, the activation mechanism of Gab2 does not make a difference what results in a logical OR connection represented by two (independent) activation arrows: Grb2→Gab1 OR EGFR→Gab1. **D** In this example, we cannot immediately decide whether both Raf-1 and MEKK1 are necessary for the activation of MKK1 (in the model description we use the synonym MEK1) or if the activation of one of these two kinases suffices. Further information is required or an ITT gate can be used (in model M1 we used an OR based on facts published in the literature).

Translating a stoichiometric model into a logical model is not a trivial task and requires additional information. Whenever a species is only influenced by one upstream molecule, the interpretation as a Boolean function is straightforward: the downstream species is active (state 1) if and only if the state of the upstream species is 1 (vice versa if the influence is negative) (see Figure 2A). In some other cases it is clear how to code the dependency in a logical function – for example, the formation of a complex (e.g. the heterodimerization of c-Jun and c-Fos to the transcription factor AP-1 (see Figure 2B) or binding of a ligand to a receptor), where all involved proteins have to be present to trigger downstream events and are thus connected with an AND gate. Furthermore, we use an OR gate whenever a protein can be recruited through different receptors or adapter proteins (see Figure 2C).

However, in many cases the stoichiometric information is not sufficient to approximate the activation level of a species as a logical function of the states of its upstream effectors and one requires additional (mainly qualitative) information, which can often be obtained from the literature.

The two main cases that can arise are the following:

- A species is positively influenced by two (or more) upstream molecules, for example a protein that can be phosphorylated by different kinases (see Figure 2D). Here, the decision whether both kinases are necessary or if one suffices, that is whether to use an AND or an OR, cannot be made on the basis of the information that is contained in a stoichiometric model. However, the necessary information can often be obtained from related literature (e.g. from knock-out studies where one of both effectors has been removed, or if an inhibitor is available for an upstream species).
- A species is positively influenced by one species (for example a kinase) and negatively influenced by another (for example a phosphatase). In this case, we cannot be sure what happens when both the kinase and the phosphatase are present; it will depend on the respective strength (described as kinetic parameters in a quantitative model) and may differ in different cell types. However, the activation of phosphatases often occurs as a temporarily secondary event upon stimulating a signaling pathway (required for switching off the signal). They may therefore be neglected when considering the early events, i.e. the initial response of the network that follows upon stimulation (see below).

We also have to keep in mind that, in all cases, the logical description is only a discrete approximation of a quantitative reaction. In those cases where neither an AND nor an OR is a good approximation, we can use incomplete truth tables [30]. This operator, herein after referred to as “ITT gate”, returns 1 if and only if all positive arguments are 1 and all negative arguments are 0, and returns 0 if and only if all positive arguments are 0 and all negative arguments are 1. In all other cases, no decision can be made and the response of the molecule remains undefined. Using ITT gates may limit the determinacy of the model (when performing stimulus-response simulations it can happen that some states cannot be determined uniquely), but it allows for a safer interpretation of the results. To illustrate this concept and to discuss uncertainties in our reconstructed logical model (in the following referred to as model M1) we consider a model variant M2 where the activation mechanisms of 14 proteins are described with ITT gates reflecting the uncertainties in the logical description of M1 (see Table S2). In this way model M2 accounts explicitly for the uncertainties in the logical concatenation of different signals, however, it cannot account for uncertainties that are captured in the wiring diagram itself.

Whenever we refer in the following to “the logical model” we refer to M1 if not stated otherwise.

Once the network construction has been completed, one may start to perform discrete simulations. We will not study the transient behavior of the network; instead we propagate the signals from the input to the output layer. Mathematically, we compute the logical steady state that follows from exposing the network to a certain input stimulus (possibly in combination with network interventions; see Methods). In this way we can analyze the qualitative input-output behavior of the network. Feedback loops, which can be identified in the interaction graph underlying the logical model, may hamper this kind of analysis of the discrete behavior of logical networks (especially negative feedback loops [30]). However, herein we will focus on the initial response of the network nodes induced by external stimulations or perturbations. Assuming that the system is in a pseudo-steady state at the beginning, the initial response of a node is governed by the paths connecting the inputs with this node whereas feedback loops are secondary events that can only be activated at a later time point when each node in the loop has exhibited its initial response. Although path/cycle length is no precise measure for the velocity of signal transduction, the comparable average length of input/output paths (19) and feedback loops (17) supports the assumption that the initial response of the network nodes is dominated by the input/output paths whereas feedback loops may overwrite the initial response of the network nodes only after a certain time period with significant length (again, feedback loops can causally not be activated before the initial response occurred). To decouple the initial response from the activity of the feedback loops, we proceed as follows: we assign to each reaction a time variable τ determining whether the reaction is active/available during the initial response (i.e. is an early event; τ = 1) or not (late event; τ = 2). In each negative feedback loop we identify the node Z that has the shortest distance to the input layer. This node Z can be considered as the initialization point of the feedback loop and we then assign τ = 2 to the “last” interaction of the feedback loop closing the cycle in node Z (i.e. points into Z). For example, in a causal chain

Input→A→B→C--|D→B we would consider D→B as a late event. In this way we interrupt the feedback loop and the logical steady states computed in the network reflect the initial response of the nodes. Strikingly, it is sufficient to consider only four interactions as late event to break all feedback loops (see below) in the network. With this acyclic network a unique logical steady state follows for any set of input values in model M1. The assignment “late” was not only reasonable for selected interactions in feedback loops, but also for three interactions involved in negative feed-forward loops down-regulating the signaling after a certain time. The time variables for each reaction can be found in Table S1. Although “late” interactions are neglected when calculating the early signal propagation, they are nevertheless important to describe structural properties of the network that can be derived from the interaction graph representation (see below). It is also important to mention that the logical steady state computed for a given scenario (see Methods) does not necessarily reflect the activation pattern in the cell at one particular point of time. Instead, it reflects for each species the initial response to the stimulus. The time range in which this initial response takes place can differ for each molecule – typically, a species situated in the upper part of the network (e.g. a receptor) responses faster to the stimulus than a species of the output layer (e.g. a transcription factor).

We set-up models M1 and M2 with *ProMoT* [31] and exported the mathematical description as well as the graphical representation to the analysis tool *CellNetAnalyzer* (*CNA*) [32]. The results obtained with *CNA* have been re-imported to and visualized in *ProMoT*.

The logical model is represented as logical interaction hypergraph (see Methods) and contains 104 nodes and 204 hyperarcs (interactions). Seven interactions are configured as late events (see Table S1), so their time scale is set to 2. Two interactions are only considered in the analysis of the interaction graph but excluded in the logical analysis as they do not change the logical function of their target node or as the exact mechanism of the interaction is unknown (see Table S1). 28 nodes are inputs of the model, i.e. their regulation is not explicitly considered in the model but can be used to simulate different scenarios. Besides ligands and receptors, these include for example some phosphatases with unknown activation mechanism. For all input nodes, a default value is given in Table S1 (and is indicated in Figure 1) that is used for the logical analyses unless otherwise specified.

### Topological properties of the interaction graph

A logical model in hypergraph form has a unique underlying interaction graph (see Methods) capturing merely positive and negative effects between the elements (instead of deterministic logic functions). Importantly, the usage of ITT gates in model M2 does not change the underlying interaction graph implying that all results obtained in this section are valid for both M1 and M2. A graph-theoretical analysis of the interaction graph enables us to derive important topological properties of the network, independently of the Boolean description. For example, the existence of feedback loops is necessary for inducing multistationarity (positive feedback loops) or oscillatory behavior (negative loops) of the dynamic system [33],[34]. In our model, the underlying interaction graph has 236 feedback loops, thereof 139 negative. Strikingly, all positive feedback loops are composed of a negative feed-forward and a negative feedback, except one that describes the reciprocal activation of the adaptor protein Gab1 and PIP3, a lipid of the membrane layer [35]. All negative feedback loops arise from five mechanisms: (i) the kinases ERK1/2 and p90RSK downregulate their own activation by phosphorylation of SOS1, a guanine nucleotide exchange factor (GEF) for Ras, (ii) the phosphatase SHP1 binds to the autophosphorylated ErbB1-homodimers and dephosphorylates them, (iii) Ras positively influences its GTPase activating protein RasGAP via PI3K, (iv) the ubiquitin ligase c-Cbl binds to ErbB1, leading to degradation of the receptor in the lysosome and (v) Ras potentiates the Rab5a-GEF activity of Rin1 and thus increases the formation of endocytic vesicles. Therefore, removing the species Ras and ErbB1-homodimer breaks all negative feedback loops. As described above, when considering the early response in the model the “last” interaction closing a feedback loop is considered as late event (see Table S1). It turned out that assigning only four interactions the “late” attribute τ = 2 suffices not only to break all negative feedback loops, but also the positive ones, so that no feedback loop remains in the network when considering the early events.

In terms of graph theory, a feedback loop is (per definition) a strongly connected subgraph, i.e. if two species *A* and *B* are part of a directed cycle it always holds that there exists a path from *A* to *B* and from *B* to *A*. In our model, all feedback loops build up one strongly connected component consisting of 34 species, meaning that all feedbacks are coupled.

**Figure 3:**
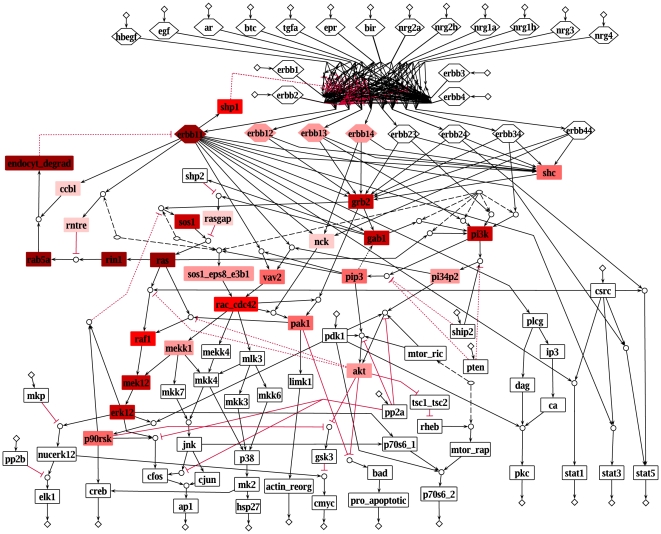
Species participation in the feedback loops. The darker a species is colored, the more loops it participates in. Colorless species are not part of feedback loops. All colored species build up one strongly connected component in the underlying interaction graph.

Figure 3 shows the participation of the different species in the feedback loops. Remarkably, the small G protein Ras is included in 98% of the loops, underlining its central role in the regulation of this network. Ras is a key regulator of cell fate [36] and a known oncogene in many human cancers [37]. However, the high number of feedbacks containing Ras in our model can also reflect the fact that Ras is one of the best studied proteins and therefore the feedback mechanisms of Ras are possibly better known than those of other proteins.

Also noteworthy, RN-tre, a GTPase activating protein (GAP) for Rab5a, is only involved in positive loops, whereas the guanine nucleotide exchange factor for Rab5a, Rin1, takes only part in negative feedbacks.

The large size of the network gives rise to a high number of possible signaling paths along which one node may affect another one. There are, for instance, 6786 paths (thereof 52% negative) leading from the input (ligand) EGF to the transcription factor AP-1 in the output layer. Considering only the early events, 1684 paths remain being 25% of them negative, where all these negative paths include the node RasGAP.

**Figure 4:**
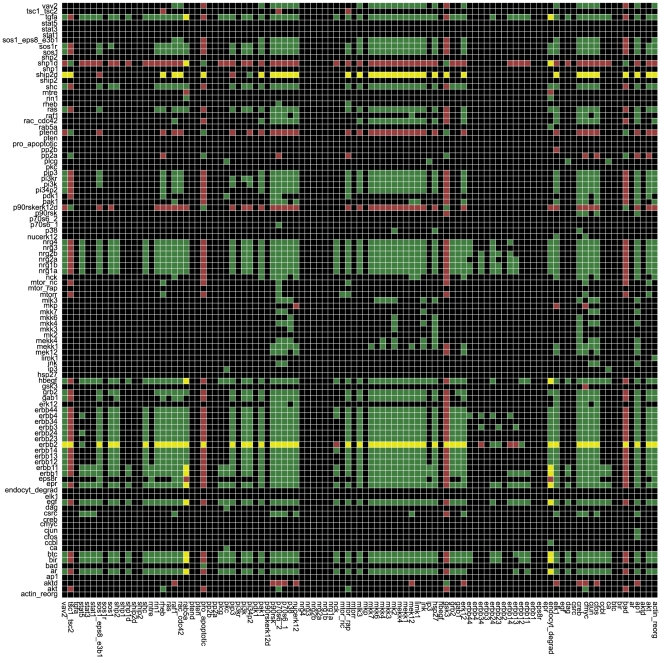
Dependency matrix D for the early events (influence of RasGAP not considered). The color of matrix element *D_i j_* means the following: green: species *i* is an activator of species *j* (there are only positive paths connecting *i* with *j*); red: *i* is an inhibitor of *j* (there are only negative paths connecting *i* with *j*); yellow: *i* is an ambivalent factor for *j* (there are positive and negative paths connecting *i* with *j*); black: *i* has no influence on *j* (there is no path connecting *i* with *j*). (See also [32]).

The information whether a species acts positively (activating) or/and negatively (inhibiting) on another species, i.e. whether there is any positive or/and negative path linking the two species, can be stored and visualized as *dependency matrix* [30]. The dependency matrix for the early events contains ambivalent dependencies (i.e. a node has positive and negative effects on other nodes) that mainly rely on the negative influence of RasGAP: as it inhibits Ras, it gives rise to a number of negative paths connecting the activated receptors with proteins downstream of Ras – in addition to the positive paths via SOS1, an activator for Ras. Not considering RasGAP leads to a matrix where only a few ambivalent interactions occur (see Figure 4): for example, the receptor ErbB2 is an ambivalent factor for almost all downstream elements as it is the preferred heterodimerization partner of the other receptors and thus prevents signaling through various different dimers (for example, ErbB1/ErbB3 formation is repressed if ErbB2 is present). When all interactions are active, the dependency matrix contains more ambivalent interactions than it does when considering only the early events.

Note that, except for ambivalent dependencies, the qualitative effect (up/down) of perturbations can be unambiguously predicted from the dependency matrix and we will make use of this technique when analyzing experimental data (see below).

### Theoretical analysis of the logical model

Implementing a Boolean function in each node of the interaction graph enables us to calculate the qualitative network response to a certain stimulus or perturbation and to predict the effects of interventions. Given the binary states for the input variables and optionally for species that have a fixed value (e.g. simulating a knock-out or knock-in), one determines the resulting logical steady state by propagating the signals according to the logical function of the nodes (see Methods).

Using this technique, we determined the network response in model M1 upon stimulation with the different ligands, again focusing on the early events (i.e. the interactions with τ = 2 were set to zero). Due to the fact that the resulting network is acyclic (as explained above), a unique logical steady state follows for any set of input values in model M1.

We found that the outputs can be divided into two groups: the majority of the output elements can be activated by all possible dimers. However, PKC, STAT1, STAT3 and STAT5 can only be activated through ErbB1-homodimers (PKC, STAT1, STAT3) or ErbB1-homodimers and ErbB2/ErbB4-dimers (STAT5). Accordingly, stimulation with neuregulins does not result in activation of the protein kinase PKC and the transcription factors STAT1 and STAT3, in contrast to stimulation with the other ligands that activate all output molecules except the pro-apoptotic effect of BAD which is repressed. This is due to the fact that the neuregulins, unlike the other ligands, do not bind to ErbB1 and thus cannot activate ErbB1-homodimers.

Strikingly, despite of the 14 ITT gates in model M2, the logical steady state in response to ErbB1-homodimers can still be determined in model M2 and does not differ from M1. This observation reflects a high degree of redundancy in at least some parts of the network. The state of each of the different kinases phosphorylating p38 or MKK4 is for example only dependent on the activity of Rac/Cdc42 so that these kinases are always activated together (see below). Thus, the input–output behavior of the network can be uniquely predicted for all ligands except neuregulins. In contrast, model M2 fails to predict the response for some nodes if other dimers (in absence of the ErbB1-homodimer) are stimulated. This concerns in particular most of the output nodes; the states of PKC, STAT1, STAT3 and STAT5 can be determined (as in model M1, these proteins can only be activated by ErbB1-homodimers, except STAT5 that is “on” in response to ErbB2/ErbB4-dimers) whereas the state of the other output nodes cannot be calculated. The indeterminacy of M2 with respect to stimulations of dimers others than ErbB1-homodimers can be explained by the uncertainty (ITT gate) in the activation of Rac/Cdc42.

When performing simulations with M1, we realized that certain species in the network show strongly coupled behavior. This guided us to search systematically for equivalence classes of network nodes whose activation pattern is completely coupled: for species *A* and *B* being elements of the same equivalence class, it either holds that their states are always the same (*A* = 0⇔*B* = 0, *A* = 1⇔*B* = 1; positive coupling) or always the opposite (*A* = 0⇔*B* = 1, *A* = 1⇔*B* = 0; negative coupling) irrespective of the chosen inputs. In other words, the state of one species in the equivalence class determines the states of all other species in this class. Hence, whenever a species of a particular equivalence class is active, we can conclude that all other species of the same equivalence class must have been activated (deactivated in case of negative coupling), at least transiently.

An algorithm to compute the equivalence classes efficiently is given in the Methods section. In general, equivalence classes can be computed for a given scenario (defined by a specific (possibly empty) set of fixed states, typically from input nodes). For this given scenario we test systematically for each species whether it is completely coupled with other nodes or not.

This type of coupling analysis is very similar to enzyme (or reaction) subsets known from metabolic networks [38],[39] and it helps to uncover functional couplings embedded in the network structure. We anticipate that the concept of equivalence classes also provides a basis for model reduction (e.g. when computing logical steady states), similar as it has been employed in metabolic networks (see e.g. [40]).

**Figure 5:**
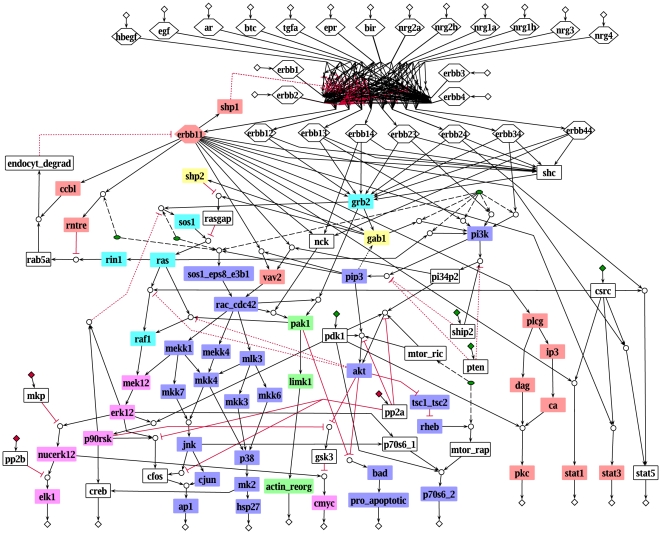
Equivalence classes in the EGFR/ErbB model. Each color represents one equivalence class. Species with no color are not part of any equivalence class. The states for the ligands and the four receptor monomers are left open, all other inputs are fixed to their default value (see Table S1), which is indicated by the red (0) and green (1) diamonds. Late events are excluded and therefore shown as dotted lines (see also figure 1).

Figure 5 shows the equivalence classes in the EGFR/ErbB model for early signal propagation where the states (presence) of all ligands and receptors were left open (the states of the other inputs were fixed to their default value as given in the model description (see Table S1)). We found six equivalence classes, the largest comprising 24 species. The latter includes parts of PI3K signaling as well as the Rac induced parts of the MAPK cascades reflecting the strong coupling of these two major pathways in model M1.

In model M2, this equivalence class splits into three smaller ones because the ITT gates introduce uncertainties that may lead to a decoupling of the two pathways. The other equivalence classes of M2 hardly differ from the ones in M1 (see Figure S1) again indicating that alternative pathways contribute rather to a higher degree of redundancy than to a higher degree of freedom regarding the potential input-output behavior.

Another concept relying on the logical description is the computation of minimal intervention sets (MIS; [30],[32]). An MIS is a set of interventions that induces a certain response, whereas no subset of the MIS does (i.e. an MIS is support-minimal). One application of MIS is to determine failure modes in the network that lead to an activation of elements of the output layer without any external stimulation of the cell. In the EGFR/ErbB model we are interested in failures that stimulate proliferation and growth of the cell when no ligand is present. Regarding the early events, constitutive activation of Ras, for example, leads to activation of the transcription factors Elk1, CREB, AP-1 and c-Myc, the p70S6 kinase, the heat shock protein Hsp27 and represses apoptosis – without any external stimulus. Besides Ras, it is sufficient to permanently activate one of the species Gab1, Grb2, PI3K, PIP3 or Shc to activate/inhibit these outputs. In model M2, the minimal intervention sets to provoke the above mentioned response contain at least two elements, for example the activation of Grb2 and Vav2.

These findings show that the network has fragile points where a mutated protein (e.g. one that is constitutively active) may support uncontrolled growth and proliferation. However, besides ErbB signaling, various other pathways are important for the regulation of growth and apoptosis and a failure in one pathway might be compensated by another, what makes it important to include these pathways step by step into our model. Additionally, when building up the model we did not focus on one certain cell type, but collected species and interactions that have been detected in different kinds of cells leading to a kind of “master model”. A model that describes only one cell type would probably include less interactions (Saez-Rodriguez *et al*, in preparation), so that a (constitutive) signal has not such a global (network-wide) influence as in the master model.

### Analyzing high-throughput experimental data

One of the strengths of our model lies in the broad range of pathways it covers and in the easy simulation of the network wide response to different stimulations and interventions. It is therefore well-suited to analyze high-throughput data where various readouts are measured in response to several stimuli and to perturbations all over the network. Here we discuss the analysis of two datasets collected in primary human hepatocytes and the hepatocarcinoma cell line HepG2. In the first set of measurements - a subset of the “CSR liver compendium” (Alexopoulos *et al*, in preparation) - primary cells and HepG2 cells were stimulated with transforming growth factor alpha (TGFα) and additionally treated with seven different small-molecule drugs, whereof six inhibit the activation of nodes considered in our model. For the second data set, HepG2 cells were stimulated with different ligands of the EGF family and treated with an inhibitor for PI3K. In both cases, the phosphorylation state of 11 signaling proteins included in the ErbB model were measured after 0, 30 and 180 minutes (see Methods for a more detailed description of the experiments). Here, we only focus on the early response of the network after 30 minutes because we want to analyze which proteins become activated at all. We assume that in hepatocytes only ErbB1 and ErbB3 are expressed as it has been reported for adult rat liver [41]; thus, for the analysis of the hepatocyte data, the state values of the other two receptors (ErbB2 and ErbB4) were set to 0 in the model.

As discussed earlier, our modeling framework is based on two concepts: (i) the Boolean (logical) description discretizing the kinetic behavior, and (ii) the underlying interaction graph reflecting the topology of interactions. This gives rise to two different approaches for the analysis of the data. First, using the dependency matrix of the interaction graph, we examined whether the experimental results are in accordance to the causal dependencies in our network. Second, using the logical model, we predicted the binary network response to the different experimental stimuli and compared these predictions with a discretized version of the data.

### Interaction graph-based data analysis

In the experiments, the phosphorylation state of the readouts is measured in response to a particular set of stimuli by adding certain ligands and/or inhibitors and combinations thereof. For each pair of treatments it can then be checked whether the ratio of the measured responses is consistent with the causal dependencies in the network topology (as captured in the dependency matrix; Figure 4) or not.

By comparing the measured phosphorylation state of a protein *p* under treatment *A*, X*^p^*(*A*), with the measured value for *p* under treatment *B*, X*^p^*(*B*), we can characterize the effect of the difference of both treatments on the activation level of *p*. We restrict ourselves here to comparing treatments that differ only in adding or removing one ligand or inhibitor, although, in principle, all possible pairwise comparisons of treatments could be considered.

As an example, assume we compare the phosphorylation state X*^p^*(*A*) of protein *p* in response to a stimulation *A*, where a ligand *l* and inhibitor *i* were added, with the state X*^p^*(*B*) of *p* in response to treatment *B*, where only the inhibitor *i* was added. An increase in the phosphorylation state of protein *p* in response to the addition of the ligand (i.e. X*^p^*(*A*)/X*^p^*(*B*)>1) indicates that there must be at least one positive path leading from this ligand to the protein and the respective entry in the dependency matrix (row *l*, column *p*) of the model should therefore show an activating or at least ambivalent influence.

Analogously, for studying the influence of a certain inhibitor, a decrease (increase) in the data in response to inhibiting a certain protein indicates that there must be at least one positive (negative) path leading from the inhibited species to the respective readout.

**Figure 6:**
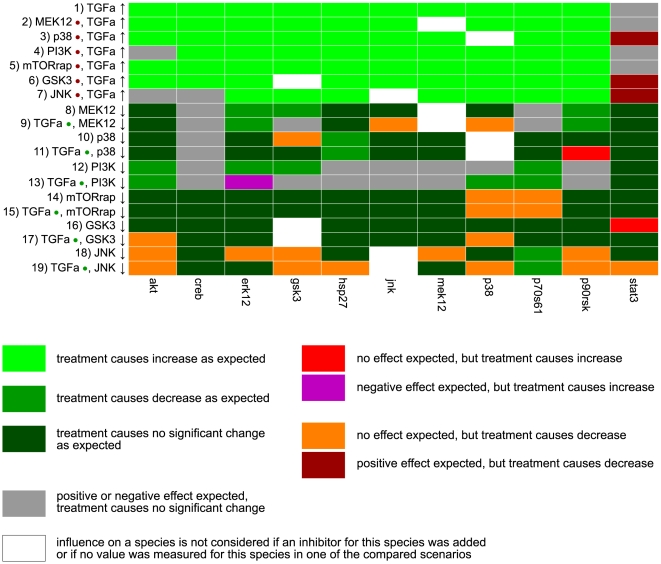
Interaction graph-based comparison between experimental data and topological properties of the model (data from primary hepatocytes). Shown is the comparison between the measured and predicted changes (“ups” and “downs”) in the activation levels of network elements in response to ligands and inhibitors in primary human hepatocytes (data obtained from Alexopoulos *et al*, in preparation). Each row compares two different scenarios *A* and *B*. A dot behind the species name in the row labels indicates that, in both scenario *A* and scenario *B*, this species was added as ligand (green dot) or an inhibitor for this species was added (red dot). Species whose input values differ in both scenarios are marked with an up or down arrow, respectively. For example, the comparison of scenario *A* (EGF ligand, TGFα ligand, PI3K inhibitor) and scenario *B* (TGFα ligand, PI3K inhibitor) is labeled by TGFα • (green dot), PI3K • (red dot), EGF ↑, i.e. the influence of an increased level of EGF on the readouts is analyzed (under the side constraints that TGFα and a PI3K inhibitor were added as well; for further explanations see text). The readouts are shown in the columns. The color indicates whether the model predictions and the measurements are consistent or not (see color legend).

**Figure 7:**
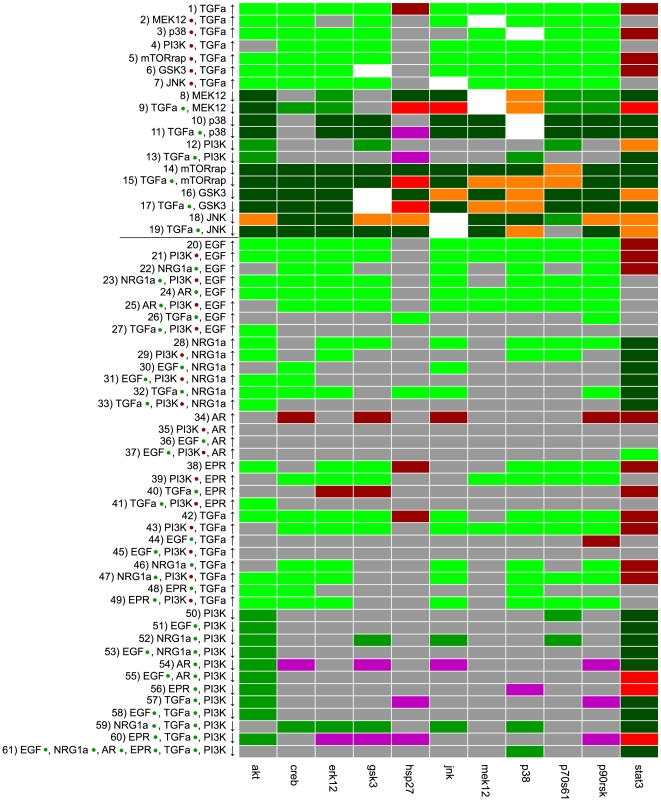
Interaction graph-based comparison between experimental data and topological properties of the model (data from HepG2 cells). Shown is the comparison between the measured and predicted changes (“ups” and “downs”) in the activation levels of network elements in response to ligands and inhibitors in HepG2 cells. The horizontal line separates the first (top) from the second (bottom) dataset for HepG2 cells (see also text). For further explanations and color legend see Figure 6.

We decided to consider a change in the data as significant if X*^p^*(*A*)/X*^p^*(*B*)>1.5 or if X*^p^*(*A*)/X*^p^*(*B*)<1/1.5. Figures 6 and 7 show the comparison of the data with the dependency matrix of the model where we considered only the early events and neglected the influence of RasGAP (as discussed above).

All in all, the experimental network response to the different treatments agrees reasonably well with the structure of the model, in particular in primary cells. In HepG2 cells, 10% of the analyzed dependencies are contradictory to our model: in 3% (7%) of the cases we saw a significant increase (decrease) in the activation level, although this was excluded by the model. 45% of the cases agreed explicitly with the model: in 28% (5%) of the cases, treatments that have a purely positive (negative) influence according to the dependency matrix resulted in a significant increase (decrease) in the measured activation levels and in 12% of the cases a ligand/inhibitor causes no significant change in a measured readout as predicted in the model. In the remaining 45% of the cases (gray entries in Figure 7), the data show no significant change, although the stimulus can affect the readout in our model (many of these gray entries will be discussed below). In primary cells, 13% of the predictions were false, 74% were fully correct and for 13% we observed no significant changes, although the model contains paths between the stimulus and the readout. A discussion of specific findings is given below together with the result of the logical model.

### Data analysis with the logical model

Whereas the dependency analysis described above is based on the raw data, a comparison of the data with the binary network response of the logical model requires a discretization of the data, the simplest being a binarization. To obtain the discretized values, we used *DataRail*, a recently introduced MATLAB toolbox that facilitates the linkage of experimental data to mathematical models [42]. It provides a variety of methods for data processing, including algorithms to convert continuous data into binary values and to create convenient data structures for the analysis in *CellNetAnalyzer*. The discretization depends on three thresholds (p1, p2, p3) which all have to be exceeded in order to discretize the measured signal to “on” [42]: the first threshold is for the *relative significance* (the ratio between the value at time 1 (in our case after 30 minutes) and the value at time 0), the second threshold ensures the *absolute significance* (ratio between the signal and the maximum value for this signal from all measurements) and the third threshold ascertains that the signal is above experimental noise. The choice of the thresholds is quite difficult as no reference data exist that define when a molecule is “on”, that is when it is sufficiently activated to induce its downstream events. Most likely, the required level of activation differs from protein to protein and from cell to cell. However, since no information on these differences is available and to avoid unnecessary degrees of freedom, we decided to define the same thresholds for all molecules and both cell types (p1 = 1.5, p2 = 0.15, p3 = 100). Figure S2 shows the sensitivities of the binarization with respect to these three parameters.

**Figure 8:**
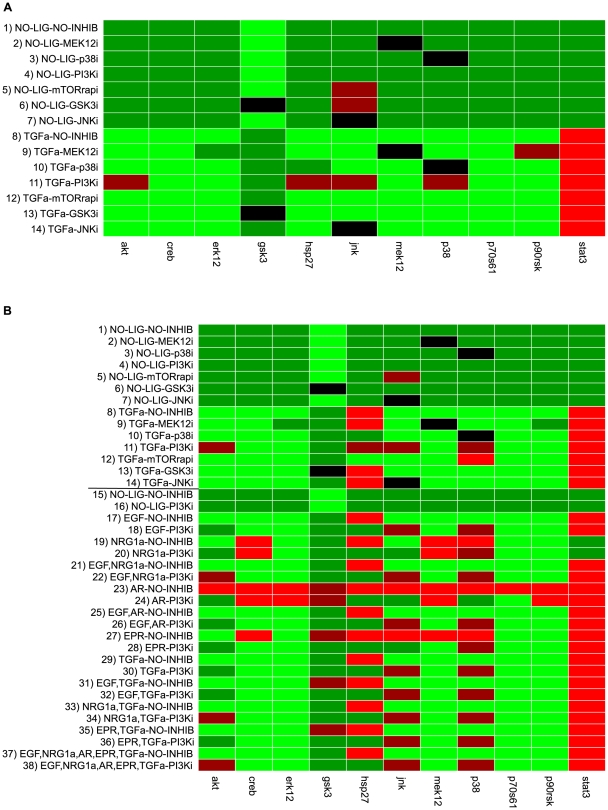
Comparison of the discretized data with predictions from the logical model. **A** Primary human hepatocytes (data from Alexopoulos *et al*, in preparation). **B** HepG2 cells (the horizontal line separates the first (top) from the second (bottom) dataset for HepG2 cells; see also text). Each row represents one treatment and the readouts are shown in the columns. Light green: predicted correctly, “on”; dark green: predicted correctly, “off”; light red: predicted “on”, measured “off”; dark red: predicted “off”, measured “on”, black: data points where the measured species is inhibited are not considered.

For each measured scenario we computed the binary network response of our model and compared it with the discretized data (Figure 8). We note that the comparison of the measured “ups and downs” with the dependency matrix (performed in the previous section) and the comparison of the discretized data with the predicted logical response are naturally correlated. However, they do not lead necessarily to exactly the same results. An example: assume you have an input stimulus (ligand L) which may activate a target species S via two independent pathways, one of both leading over an intermediate species A for which we have an inhibitor I. If we compare the scenario “stimulation with L and adding inhibitor I” against “stimulating with L” via dependency analysis we would expect a decrease in the (non-discretized) activation level of S since the inhibited species A is an activator for S. However, the phosphorylation state of S might show no significant change in the dependency analysis (i.e. leads to a “gray entry” as in Figures 6 and 7) due to the alternative pathway not affected by the inhibitor. In contrast, if the two pathways from L to S are OR-connected in the logical model, the latter would still predict S to be “on”. Another difference in the data analysis based on dependency matrix vs. logical model is that the former compares species states obtained from two different experiments (e.g. experiment with/without inhibitor) whereas the logical model gives for each experiment one (independent) prediction for each species.

As in the case of the dependency analysis, the measured data agree reasonably well with the predictions of the model M1 (HepG2: 77% correct predictions; primary cells: 90% correct predictions).

In Figure S3, the comparison of model M2 with the experimental data is shown. For primary cells, only 7% of the states cannot be determined due to the ITT gates, for HepG2 21%. 83% of the predictions for primary cells and 59% for HepG2 were correct. In all cases where a state can be predicted by M2 it naturally coincides with the prediction from M1 since the latter is only one special case of all possible behaviors in model M2.

In some cases where we used an ITT gate in model M2, the logical function can be uniquely determined with the experimental results confirming some of the deterministic logic gates used in model M1: for example, the transcription factor CREB can be activated through the MEK-dependent kinase p90RSK AND/OR through the p38 dependent MK2. As CREB is still activated both with MEK inhibitor and with p38 inhibitor, this points to an OR-connection achieving a match between model predictions and data in this node. In the same way, we can verify an AND connection for the two negative modulators of Gsk3 and an OR for the phosphorylation of the auto-inhibitory domain of p70S6 kinase.

Again, using ITT gates, we can only reflect uncertainties regarding the logical combination of different paths and not whether a species influences another at all. This is why some of the discrepancies between the predictions of model M1 and the data also appear for model M2.

### Interpreting inconsistencies between data and model predictions

Most disagreements between model predictions and experimental results concentrate on certain experimental conditions (rows) and readouts (columns) - in the dependency analysis as well as in the analysis with the logical model. Here we discuss such systematic inconsistencies and – using our model – we seek to provide explanations and conclusions:

- A significantly increased state of phosphorylation of STAT3 in response to any of the ligands could not be found both in HepG2 and primary hepatocytes. Whether this is due to the fact that the activation of STAT3 is very transient, as it has been reported for example for the human epithelial carcinoma cell line A431 [43], or if the activation of this transcription factor through ErbB receptors plays no role in hepatocytes, has still to be clarified.
- Both analysis approaches show that stimulation of HepG2 cells with amphiregulin (not measured in primary cells) did not result in activation of the measured proteins (see Figure 7, lines 34–37 and Figure 8B, lines 23/24). This is in agreement with findings of amphiregulin being a much weaker growth stimulator than EGF in some cell types [44].
- The systematic errors in the column of p38 in the dependency analysis (for primary as well as HepG2 cells) might indicate missing edges in the model requiring further experimental studies to verify these findings. We cannot exclude that other (e.g. stress-induced) pathways not captured in our model may have caused these observations, also because some of the effects on p38 are also present without ligand stimulation.
- Stimulating the HepG2 cells with both TGFα and EGF does not result in a significantly higher activation level of the readouts compared to adding only one of these ligands as can be seen from the predominantly gray entries in lines 26/27 and 44/45 in Figure 7. This finding is in accordance with the fact that both ligands are very similar and bind to the same receptor dimers (see Table S1).
- One of the major differences in the behavior of the two cell types is the activation of Hsp27: whereas this heat shock protein becomes activated in response to cytokine stimulation in primary cells, no significant increase in the state of phosphorylation occurs in almost all studied scenarios in the cancer cell line (leading to many false “on” predictions).
- Another remarkable discrepancy between the experimental data and our model predictions is the influence of the mTOR inhibitor rapamycin on phosphorylation of p70S6 kinase (see lines 14/15 in Figures 6 and 7), which is not supported by our model. Although mTOR mediates the phosphorylation of the catalytic site T389 [45], it has to the best of our knowledge not been implicated with the phosphorylation of T421 and S424, those sites, whose state of phosphorylation were measured in the analyzed data sets. However, an inhibitory effect of rapamycin on these sites has been reported earlier [46], even if the molecular mechanism that could explain this influence still has to be uncovered.
- According to our model, PI3K should influence all measured readouts except STAT3. However, the data show a clear effect of the PI3K inhibitor only on the phosphorylation of Akt (see Figure 6, lines 12/13 and Figure 7, lines 50–61). Additionally, Figure 8 shows that JNK, p38 and, in primary cells also Hsp27, could be activated in the experiments in presence of PI3K inhibitor although our model predicted the phosphorylation to be blocked (due to the AND connections of the PI3K-dependent nodes PIP3 and PI(3,4)P2, respectively, with Vav2 and SOS1_Eps8_E3b1). We therefore searched for hypothetical changes in our model structure that could explain these experimental findings. We observed that node Rac/Cdc42 lies on all paths connecting the inputs (ligands) with the aforementioned critical readouts (except Gsk3, see below), i.e. activation of Rac/Cdc42 is necessary in our model for phosphorylation of JNK, Hsp27 and p38. We may thus hypothesize that - in contrast to the assumption in our model - PI3K activity is not necessary for activation of the small G-proteins Rac and Cdc42 in primary hepatocytes and in HepG2 cells.
  A closer look on Figure 8B (lines 19/20) reveals that the phosphorylation of JNK in response to neuregulin is – in contrast to the response to any of the other ligands – sensitive on PI3K inhibitor. This is also reflected in Figure 7 where an increase of neuregulin only increases the phosphorylation of JNK in absence of PI3K inhibitor (see lines 28–33) and decreasing the level of PI3K (i.e. adding the inhibitor) after neuregulin stimulation also leads to a decreased phosphorylation state of JNK (see lines 52 and 59). Therefore, neuregulin must use a different, PI3K dependent signaling path for activating JNK than the other ligands, probably due to the fact that neuregulin only activates ErbB1/ErbB3-dimers whereas EGF, TGFα, amphiregulin and epiregulin additionally activate ErbB1-homodimers. Taking these findings together, we propose the following alternative mechanism: Vav2 is the major GEF for Rac/Cdc42 in hepatocytes and activates Rac/Cdc42 in a PI3K-independent way. Neuregulin, which cannot bind to ErbB1-homodimers and accordingly is not able to activate Vav2 (see Table S1), provokes the activation of JNK independently of the Rac/Cdc42 induced MAPK cascade through a different, PI3K-dependent pathway.
- In the model, the inhibitory phosphorylation of Gsk3 can be induced by a MEK1/2 dependent pathway (via p90RSK) and by a PI3K dependent pathway (via Akt). Figures 6 and 7 (lines 9 and 13) show that the phosphorylation of Gsk3 in response to TGFα is independent of the MEK inhibitor and the PI3K inhibitor, both in HepG2 and in primary cells. As TGFα stimulation leads to a strong phosphorylation of Gsk3 in both cell types (see Figure 8), there must be another signaling route, not involving MEK and PI3K. One possible candidate is PKC which has already been reported to inhibit Gsk3, however not in response to ligands of the EGF family [47].
- According to the data, both Gsk3 and p90RSK are influenced by JNK inhibitor after TGFα stimulation in primary hepatocytes (see Figure 6, line 18). This seems to support another possible mechanism, where JNK activates p90RSK which may then phosphorylate Gsk3. However, the JNK inhibitor affects much more proteins than expected, both in HepG2 and in primary cells. As these unexpected influences also occur in absence of ligand stimulation, this strongly suggests a minor specificity of the JNK inhibitor.
- Similar as for Gsk3 phosphorylation, data analysis with our model provides useful insights into the activation mechanism of CREB in response to TGFα: the proposed effect of the p38 dependent kinase MK2 on CREB cannot be observed both in HepG2 and in primary cells (see Figures 6 and 7, line 11). The positive effect of MEK on CREB phosphorylation after TGFα stimulation can be seen in HepG2 (Figure 7, line 9), but not in primary hepatocytes (Figure 6, line 9). Together with the finding of the logical analysis that the MEK inhibitor cannot block activation of CREB in HepG2 (Figure 8), this indicates that there must be an alternative pathway for CREB activation in primary hepatocytes that is probably involving p90RSK.

A summary of the above mentioned results is given in Table S3. Changing the model accordingly, we can improve the agreement of model predictions and data in the logical analysis from 90% to 97% for the primary cells and from 74% to 94% for HepG2. For the dependency analysis, the number of comparisons that agree explicitly increases from 74% to 82% for primary and from 45% to 64% for HepG2 cells. Moreover, the number of entries where we assumed a change in the data but could not detect a significant increase or decrease reduces from 13% to 4% (primary) and from 45% to 24% (HepG2), albeit at the expense of a minor increase in the number of contradictions (primary: increase from 13% to 14%, HepG2: 10% to 12%).

As described above, herein we deduced the proposed changes of the model structure manually from the data analysis. More systematic approaches for network identification from combinatorial experiments are given in Saez-Rodriguez *et al* (in preparation) and in [48].

In general, detecting such systematic inconsistencies of the data both with respect to the dependency structure of the network and the logical model description is a great advantage of our approach and could hardly be achieved with a model relying on differential equations (where parameter uncertainty often hampers a falsification of the model structure).

## Discussion

In the present work, we developed a large-scale logical model of signaling through the four ErbB receptors, including the ERK, JNK and p38 MAPK cascades, Akt signaling, activation of STATs and the PLCγ pathway, based on the stoichiometric pathway map of Oda *et al* [14]. We discussed technical problems that arise when converting a stoichiometric model into a logical one and proposed a general guideline how to deal with them.

We examined several properties of the logical model characterizing its topology (feedback loops and network-wide interdependencies as derived from the underlying interaction graph) and its qualitative input-output behavior with respect to different stimuli. We also introduced the new technique of species equivalence classes revealing coupled activation patterns in the logical model providing valuable insights into the correlated behavior of network elements.

One possibility to deal with uncertainties concerning the correct logical combination of different influences on a certain node is the usage of gates with incomplete truth tables (ITT gates). We replaced the (deterministic) logical gates for the activation of 14 species of our model with ITT gates and repeated all logical analyses with this modified model. Surprisingly, the predictive power of the ITT model is still high, highlighting the redundant structure of major parts of the signaling pathway and showing that many properties of the network do not rely on the assumptions we made when choosing the logical functions.

Compared with a dynamic model based on differential equations, our approach for describing signaling events is certainly limited in reflecting kinetic aspects which are important to obtain a complete understanding of these processes in the cell. However, properties derived exclusively from the structure can provide insights into the transfer of signals in the cell, as the result of this and other studies have shown [28],[29]. The simpler design of the qualitative models also has some advantages over complex dynamic models. First of all, the logical approach enables us to model large-scale signaling networks allowing, for example, to study the effects of crosstalk, for which a dynamic description is currently often unimaginable. An expansion of the model can easily be done, whereas adding a reaction to a model of differential equations requires usually the elaborate re-estimation of parameters. The flexible architecture of the model also enables us to test and generate hypotheses very quickly. Another advantage is that the qualitative predictions derived with a logical model do not depend on certain parameter values except the time scales and are therefore more generally valid. There are also methods to study ODE models without parameters (e.g. [49]–[51]). However, these methods are currently limited to relatively small systems and study different properties.

With the advances of experimental techniques, it becomes more and more essential to provide tools that allow for the analysis and exemplification of the huge amount of data that arise. We developed new techniques for the analysis of large data sets that are especially well-suited to analyze data that stem from combinatorial experiments (systematic combination of different ligands/inhibitors). The first approach, a method for comparing experimental (high-throughput) data with predictions derived from the logical model, requires a discretization of the data. Although the “on/off” decision is sometimes hard to take as no reference data exist and the “right” thresholds for the parameters are unknown, assessing the sensitivities of the data with respect to the discretization thresholds leads to a safer interpretation. Alternatively, the data can be assigned a relative value between 0 and 1 which can be compared to the discrete (0/1) value of the model (Saez-Rodriguez *et al*, in preparation). The second approach, the comparison of the data with the topological dependency structure of the model (captured in the interaction graph), requires only a significance threshold and provides an even simpler method for the falsification of qualitative knowledge as it relies on less assumptions than the logical model (only the wiring diagram is evaluated; logical combinations and discrete states are not required).

Applying these new automatized techniques to analyze high-throughput phospho-proteomic data revealed some important insights into the structure of EGFR/ErbB signaling in primary hepatocytes and the HepG2 cell line. Our results strongly suggest a model where the Rac/Cdc42 induced p38 and JNK cascades are independent of PI3K, both in primary hepatocytes and in HepG2. Furthermore, we detected that the activation of JNK in response to neuregulin follows a PI3K-dependent signaling pathway that seems not to be important for activation of JNK through ErbB1-binding ligands. Additional findings concern Gsk3 and CREB where known signaling paths were excluded to provoke phosphorylation after TGFα stimulation and new routes could be proposed. Finally, we observed no activation of STAT3 in both cell types and no activation of Hsp27 in HepG2. Besides these results on the topology of EGFR/ErbB signaling in hepatocytes, the comparison of model predictions and data could also detect side effects of the used JNK inhibitor.

With our software *CellNetAnalyzer* (*CNA*; [32]) we provide a powerful tool to study structural networks. It facilitates the analysis of interaction graphs as well as logical models and also provides methods to compare model predictions with experimental data as described herein. Furthermore, *CNA* is now highly coupled with the tools *ProMoT* [31], *DataRail* [42] and *CellNetOptimizer* (Saez-Rodriguez *et al*, in preparation), forming an integrated pipeline for the construction, structural analysis and data interpretation of signal transduction networks.

The presented model is to the best of our knowledge one of the largest existing mathematical models of the EGFR/ErbB signaling pathway. However, it is far from being complete and has to be complemented, for example by including the endocytosis of the receptors. Step by step, we want to expand the model by other important mitogenic and pro- and anti-apoptotic pathways to study crosstalk. We also think that the logical model can serve as a useful basis for the development of dynamic models. A step between both modeling frameworks could be to refine the current binary description and use multilevel activation instead, a promising approach yet it requires more detailed (semi-quantitative) information on the reaction kinetics and leads to more complex networks. Further refinements could be achieved by fuzzy logic description or by considering more precise time delays for the interactions.

## Methods

### Logical modeling of the EGFR/ErbB signaling network

For the reconstruction and qualitative analysis of the EGFR/ErbB signaling network we employ a logical modeling framework as introduced previously [30],[32]. Signaling networks are usually structured into input, intermediate and output layer and the input signals govern the response of the network. For this characteristic network topology we introduced *logical interaction hypergraphs* (LIHs) as a special representation of Boolean networks, which is well-suited to formalize, visualize and analyze logical models of signal transduction networks. As in all Boolean networks, nodes in the network represent species (e.g. kinases, adaptor molecules or transcription factors) each having an associated logical state (in the binary case as used herein only “on” (1) or “off” (0)) determining whether the species is active (or present) or not. Signaling events are encoded as Boolean operations on the network nodes. For example, the MAP kinase (MAPK) JNK can be activated (gets “on”) if the MAPK kinase MKK7 AND the MAPK kinase MKK4 are active (see the AND connection in Figure 1). Usually, a node can be activated by more than one signaling event; all these events are then OR-connected, e.g. the MAPK p38 becomes active if MKK3 OR MKK4 OR MKK6 is active (Figure 1).

In general, in LIHs we make only use of the Boolean operators AND (·), OR (+), and NOT (!), which are sufficient to represent any logical relationship. A signaling event (or interaction) in an LIH is an AND connection of nodes (negation of node values using the NOT operator are allowed) describing one opportunity how the target species of this connection can be activated. Hence, for the first example described above we would write
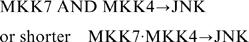
In a graphical representation of the network (see JNK node in Figure 1), such an AND connection is displayed as a *hyperarc*. In contrast to arcs in graphs, a hyperarc (in hypergraphs) may have several start or end nodes. Clearly, in some cases, only one species is required to activate another, as in the example
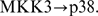
In these cases, the hyperarc is a simple arc as occurring in graphs; we will nevertheless refer to it as a hyperarc. As already mentioned, a species may be activated via several distinct signaling events (hyperarcs), i.e. all these signaling events are OR-connected. This can again be illustrated by p38, which can be activated (independently) via three different MAPKs and we therefore have three different OR-connected hyperarcs:
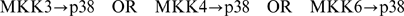
Hence, all hyperarcs pointing into a species are OR connected. In this way we can easily interpret Figure 1, which displays graphically the interactions given in Table S1.

As described in the main part, the reconstruction of our logical model of EGFR/ErbB is based on a stoichiometric model of EGF receptor signaling [14] and additional information from the literature. Some general remarks on how a stoichiometric network can be translated into a logical one are given in the main part. The logical model (for both version M1 and version M2; the latter having 14 gates with incomplete truth tables; see main text) comprises signaling of 13 members of the EGF ligand family through the EGF receptor and its heterodimerization partners ErbB2-4, leading to the activation of various transcription factors and kinases that effect proliferation, growth and survival (Figure 1). In addition to ligands and receptors, species whose regulation is not known are herein considered as members of the input layer, for example the phosphatases PTEN and SHIP2.

The differentiation between “early” and “late” events (see below and main part) makes it sometimes necessary to introduce auxiliary (“dummy”) nodes that have no biological correspondents. Consider for example a species C that is activated by species A during the early events (τ = 1) and down-regulated by another species B as a late event (τ = 2). Assuming that both the presence of A and the absence of B are necessary to activate C, we use an AND connection in the LIH representation (A · !B→C). As the two influences are combined to one hyperarc in the LIH, we can assign only one time variable to this interaction. In order to reflect the time delay of the inhibitory activity of B, we introduce an additional *dummy* node with τ = 2. We now describe the original interaction A · !B→C with two interactions
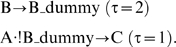
An example in the ErbB model are the ErbB1-homodimers that are activated by various ligands (e.g. EGF) and dephosphorylated by SHP1 (see Table S1). To properly describe the timing of the SHP1-mediated dephosphorylation of the receptor, we introduce a dummy species shp1d that is activated by SHP1 and obtain thus two hyperarcs:
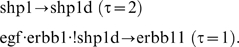
Another type of node that is introduced for modeling purpose only is what we refer to as *reservoir*. It is used whenever a molecule causes different downstream events depending on how it is activated. Here, we have to use more than one compound to describe the molecule in the model. An example in our model is mTOR: associated with Rictor, it is involved in the activation of Akt, whereas the Raptor-bound form activates p70S6 kinase. However, as all these compounds represent the same biological species, we associate them with a reservoir, pointing out that they share the same pool. Inactivation of the reservoir will then affect the activation of all correspondents of this species.

A full description of the model M1 with all species and interactions (hyperarcs) is given in Table S1. In model variant M2, 14 logical gates of model M1 have been configured as incomplete truth tables (ITT gates). The differences between M1 and M2 are described in Table S2.

### Analysis of the logical model

Once an LIH has been set-up, we may start to analyze it. A typical scenario is that we apply a pattern of inputs to the network and we would like to know how the nodes in the network will respond to this stimulation. As explained in [32], by propagating input signals along the logical (hyperarc) connections (which is equivalent to computing the logical steady state resulting from the input stimuli) we obtain the qualitative response of the network. Note that the logical steady state obtained by this propagation technique is independent of the assumption of synchronous or asynchronous switching which is required when analyzing the discrete dynamics of Boolean networks [27]. It depends on the functionality of positive or negative feedback loops in the network whether we can resolve a complete and unique logical response of all nodes for a given set of input stimuli (for example, negative feedback loops may prevent the existence of a logical steady state). Feedback loops are usually present in signaling networks, however, as described in the main part, we identified one interaction in each loop that can be considered as a late event (τ = 2). When considering the initial response of the network we set these late-event connections inactive leading to an acyclic network for which always a unique network response for a given set of inputs can be computed.

One can also easily perform *in silico* experiments, for example check how a knock-out (or inhibition) alters the network response by fixing the state of the respective species.

With the idea of minimal intervention sets (MIS) one may even directly search for those interventions that enforce a desired response (e.g. activation or inactivation of a transcription factor). As described in [32], MISs can be computed by testing systematically which combinations of knockouts and knockins fulfill a specified intervention goal.

### Species equivalence classes in logical networks

A new analysis technique for logical networks is introduced in this work: we search for equivalence classes of network nodes whose activation pattern is completely coupled in logical steady state: species *A* and *B* are elements of the same equivalence class, if it either holds that their values in steady state are always the same (*A* = 0⇔*B* = 0, *A* = 1⇔*B* = 1; positive coupling) or always the opposite (*A* = 0⇔*B* = 1, *A* = 1⇔*B* = 0; negative coupling) irrespective of the chosen inputs (e.g. ligands). In other words, the state of one species in the equivalence class determines the states of all other species in this class. Again, the relation given above holds for logical steady states where both A and B are determined and where no intervention was made in the network except for the inputs.

Whenever a species of a particular equivalence class is active, we can conclude that all other species of the same equivalence class must have been activated (deactivated in case of negative coupling), at least transiently.

An efficient algorithm for computing the equivalence classes can be constructed as follows:

1. Equivalence classes can be computed for a given scenario, so we first define a specific (possibly empty) set of fixed states, typically from (some) input nodes.
2. For this given scenario we test systematically for each species whether it is strongly coupled with other nodes or not, independently of external stimuli. For each species *A* we compute (i) the logical steady states of all other species that result when fixing the state of *A* to 1 and (ii) the logical steady states of all other species that result when fixing the state of *A* to 0. A node *B* whose logical steady state can be determined in both cases and is 1 in one case and 0 in the other case is known to be in one equivalence class with species *A*: *B* is positively coupled with *A* if the two resulting logical steady states of *B* are 1/0 (it then holds *A* = 1 = >*B* = 1, *A* = 0 = >*B* = 0 and thus according to contraposition also *B* = 0 = >*A* = 0, *B* = 1 = >*A* = 1) and negatively coupled if the two logical steady states are 0/1 (it then holds *A* = 1 = >*B* = 0, A = *1* = >*B* = 0 and thus according to contraposition also *B* = 0 = >*A* = 1, *B* = 1 = >*A* = 0). The case that the logical steady state of a species *B* is 0/0 or 1/1 (for fixing *A* = 1/*A* = 0) indicates that this species *B* can never be activated or never be inhibited, respectively, and would thus indicate a semantic problem in the model.

If a species *A* is coupled with species *B*, and species *B* is coupled with species *C*, we can subsume all three species in one equivalence class (we do that systematically for all species until we reach finally the equivalence classes). Composing the equivalence classes in this way, it may also happen that species that cannot influence each other (no directed path between both exists) are in one equivalence class due to a common upstream regulator. Consider a network that only contains the interactions A → B and A → C. Fixing the state of B or C to 1/0 we cannot conclude any equivalence relations as no further states can be determined. Fixing A to 1 and 0 we find that A is equivalent to B and A is equivalent to C, thus – according to the rule given above – A, B and C form one equivalence class.

### Interaction graph analysis

Another advantage of LIHs is that we can easily derive the (signed and directed) interaction graph underlying the logical model: we only have to split all hyperarcs that have two or more start nodes (i.e. the AND connections) into simple arcs. Interaction graphs cannot be used to give on/off predictions; however, they provide an appropriate formalism to search for signaling paths and feedback loops. Another useful feature that can be extracted from interaction graphs is the *dependency matrix* as introduced in [30],[32] which displays network-wide interdependencies between all pairs of species. For example, a species A is an activator (inhibitor) of another species B, if at least one path leads from A to B and if all those paths are positive (negative). This kind of information can be very useful for predicting effects of perturbations.

### Model implementation and availability

We set-up the logical EGFR/ErbB model with *ProMoT* [31] and exported the mathematical description as well as the graphical representation to the analysis tool *CellNetAnalyzer (CNA)* [32]. The results obtained with *CellNetAnalyzer* have been partially re-imported to and visualized in *ProMoT* (Figures 1, 3, 5). Data management and discretization was performed with DataRail [42].

The tools are freely available (for academic use) from the following web-sites:

*DataRail*: http://code.google.com/p/sbpipeline/wiki/DataRail

*ProMoT*: http://www.mpi-magdeburg.mpg.de/projects/promot/

*CellNetAnalyzer*: http://www.mpi-magdeburg.mpg.de/projects/cna/cna.html

After acceptance, the model will be provided in formats for *ProMoT* and *CellNetAnalyzer*.

### Experimental set-up and measurement data

The data on primary human hepatocytes and the first part of the HepG2 data were obtained from experiments conducted by Alexopoulos *et al* (in preparation), while for the second part of the HepG2 data, a cue-signal-response (CSR) compendium was created for the EGFR pathway. The second dataset comprises 11 phosphoprotein measurements under 24 different perturbations generated by the combinatorial co-treatments with a diverse set of ErbB ligands and the PI3K inhibitor. For ligands we choose 5 ErbB related cytokines, namely epidermal growth factor (EGF), neuregulin 1 (NRG1; also known as heregulin), amphiregulin (AR), epiregulin (EPR), and transforming growth factor alpha (TGFα). For each stimulus, the PI3K inhibitor *ZSTK*-474 was added at 2 µM final concentration 30 minutes prior to any ligand treatment. Optimal inhibitor concentration was obtained for concentration-inhibition curve (data not shown) in order to achieve 95% inhibition of the downstream pAkt signal on TGFα stimulated HepG2. The dataset was created using a high-throughput method of bead-based fluorescent readings (Luminex, Austin, TX). Assays were optimized for multiplexability and checked for passage-to-passage and preparation-to-preparation variability (Alexopoulos *et al*, in preparation).

The full dataset (first and second part) and the resulting discretization are graphically depicted in Figure S4.

## Supporting Information

Figure S1 Equivalence classes for model M2. Each color represents one equivalence class. The equivalence classes of model M1 are depicted by the species border color. Late interactions (τ = 2) are drawn as dotted lines. The value of fixed inputs is given by the green (1) and red (0) diamonds.
(0.33 MB PDF)

Figure S2 Sensitivities of the binarization to the chosen parameters. 2.1 Primary human hepatocytes 2.2 HepG2 cells (the horizontal line indicates the first (top) and the second (bottom) measurement set for HepG2 cells); Parameter p1 (2.1a, 2.2a): the ratio between the value at time 1 and the value at time 0 lies beneath (red) or above (green) the fixed threshold p1 = 1.5; Parameter p2 (2.1b, 2.2b): the ratio between the signal and the maximum value for this signal from all measurements lies beneath (red) or above (green) the fixed threshold p2 = 0.15; Parameter p3 (2.1c, 2.2c): the signal lies beneath (red) or above (green) the fixed threshold for experimental noise (p3 = 100). For all parameters: The darker a field is colored, the larger is the distance to the chosen threshold, i.e. the binarization is less sensitive on the parameter.
(0.61 MB PDF)

Figure S3 Comparison of the discretized data with predictions from model M2. A Primary human hepatocytes (data from Alexopoulos et al, in preparation). B HepG2 cells (the horizontal line separates the the first (top) from the second (bottom) dataset for HepG2 cells; see also text). Each row represents one treatment and the readouts are shown in the columns. Light green: predicted correctly, “on”; dark green: predicted correctly, “off”; light red: predicted “on”, measured “off”; dark red: predicted “off”, measured “on”; yellow: state cannot be determined in logical steady state analysis; black: data points where the measured species is inhibited are not considered.
(0.21 MB PDF)

Figure S4 Data plots generated with DataRail. Shown are the phosphorylation states of the proteins after 0, 30 and 180 minutes. Green: significant activation after 30 minutes (according to the chosen parameters); gray: no significant activation (cf. also Saez-Rodriguez et al, 2008). A Primary human hepatocytes (data obtained from Alexopoulos et al (in preparation)) B HepG2 cells, first set of experiments (data obtained from Alexopoulos et al (in preparation)) C HepG2 cells, second set of experiments.
(0.28 MB PDF)

Table S1 Logical EGFR/ErbB model: list of species and interactions.
(0.16 MB PDF)

Table S2 Incomplete truth tables (ITTs) in the model variant M2.
(0.01 MB PDF)

Table S3 Proposed model changes to improve the fit of the model to the data.
(0.01 MB PDF)
