# Supplementary material for: The Logic of EGFR/ErbB Signaling: Theoretical Properties and Analysis of High-Throughput Data
Source: PLoS Comput Biol. 2009 Aug 7;5(8):e1000438. doi: 10.1371/journal.pcbi.1000438 (PMC2710522; doi:10.1371/journal.pcbi.1000438)

Figure S1. Equivalence classes for model M2.

Each color represents one equivalence class. The equivalence classes of model M1 are depicted by the species border color. Late interactions (τ=2) are drawn as dotted lines. The value of fixed inputs is given by the green (1) and red (0) diamonds.

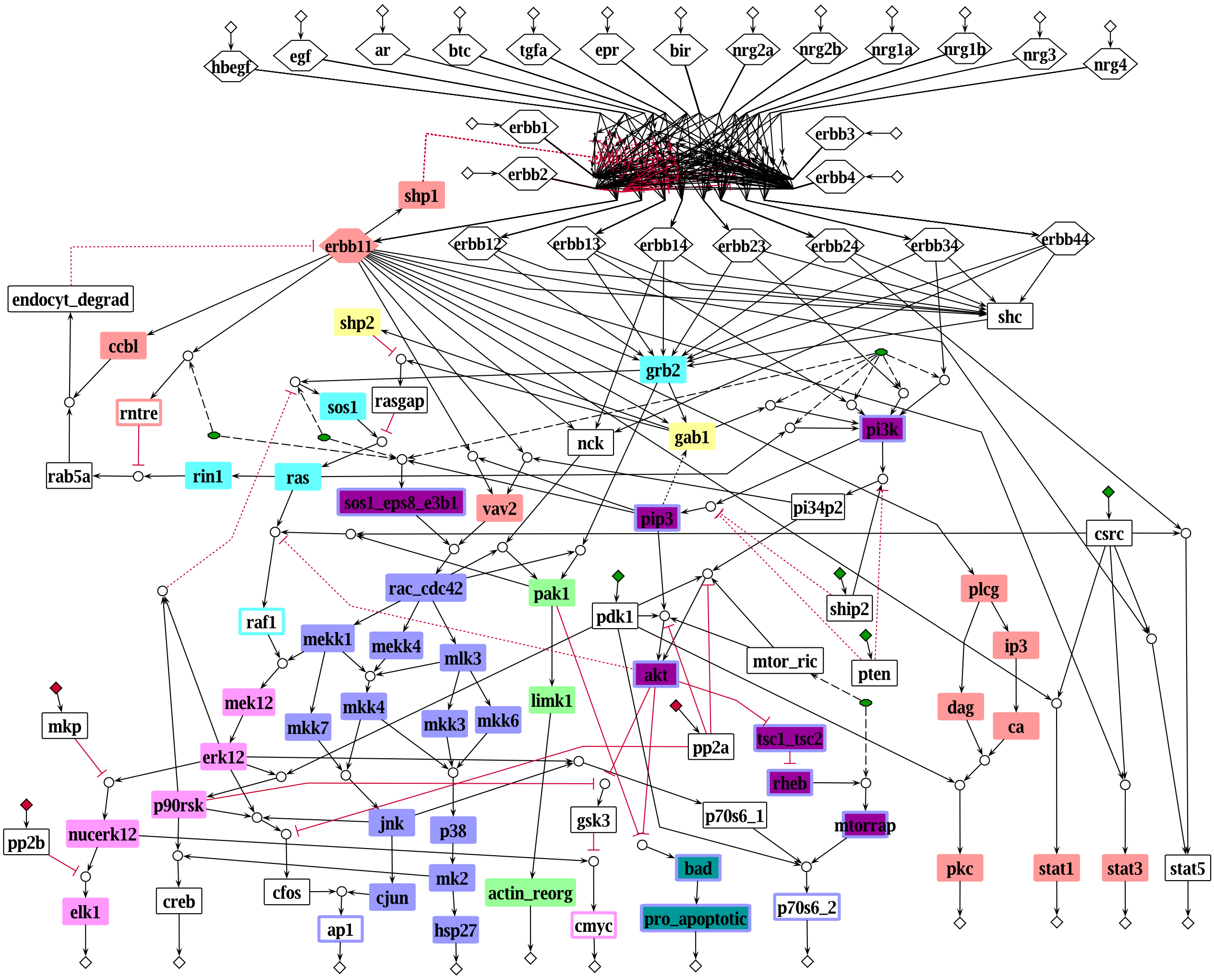

Supplement: Figure S1 — Equivalence classes for model M2. Each color represents one equivalence class. The equivalence classes of model M1 are depicted by the species border color. Late interactions (τ = 2) are drawn as dotted lines. The value of fixed inputs is given by the green (1) and red (0) diamonds. (0.33 MB PDF) [file pcbi.1000438.s001.pdf]
