# Supplementary material for: The Logic of EGFR/ErbB Signaling: Theoretical Properties and Analysis of High-Throughput Data
Source: PLoS Comput Biol. 2009 Aug 7;5(8):e1000438. doi: 10.1371/journal.pcbi.1000438 (PMC2710522; doi:10.1371/journal.pcbi.1000438)
Supplement: Figure S2 — Sensitivities of the binarization to the chosen parameters. 2.1 Primary human hepatocytes 2.2 HepG2 cells (the horizontal line indicates the first (top) and the second (bottom) measurement set for HepG2 cells); Parameter p1 (2.1a, 2.2a): the ratio between the value at time 1 and the value at time 0 lies beneath (red) or above (green) the fixed threshold p1 = 1.5; Parameter p2 (2.1b, 2.2b): the ratio between the signal and the maximum value for this signal from all measurements lies beneath (red) or above (green) the fixed threshold p2 = 0.15; Parameter p3 (2.1c, 2.2c): the signal lies beneath (red) or above (green) the fixed threshold for experimental noise (p3 = 100). For all parameters: The darker a field is colored, the larger is the distance to the chosen threshold, i.e. the binarization is less sensitive on the parameter. (0.61 MB PDF) [file pcbi.1000438.s002.pdf]

Figure S2. Sensitivities of the binarization to the chosen parameters.

2.1 Primary human hepatocytes (data obtained from Alexopoulos et al (in preparation));

2.2 HepG2 cells (the horizontal line indicates the first (top) and the second (bottom) measurement set for HepG2 cells);

Parameter 1 (2.1a, 2.2a): the ratio between the value at time 1 and the value at time 0 lies beneath (red) or above (green) the fixed threshold p1=1.5;

Parameter 2 (2.1b, 2.2b): the ratio between the signal and the maximum value for this signal from all measurements lies beneath (red) or above (green) the fixed threshold p2=0.15;

Parameter 3 (2.1c, 2.2c): the signal lies beneath (red) or above (green) the fixed threshold for experimental noise (p3=100).

For all parameters: The darker a field is colored, the larger is the distance to the chosen threshold, i.e. the binarization is less sensitive on the parameter.

2.1a)

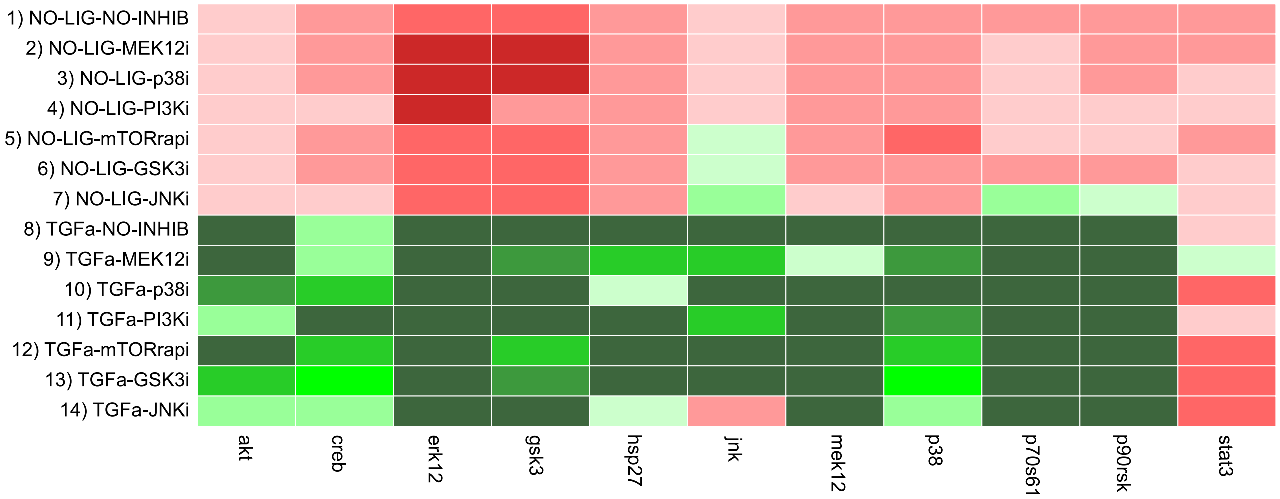

2.1b)

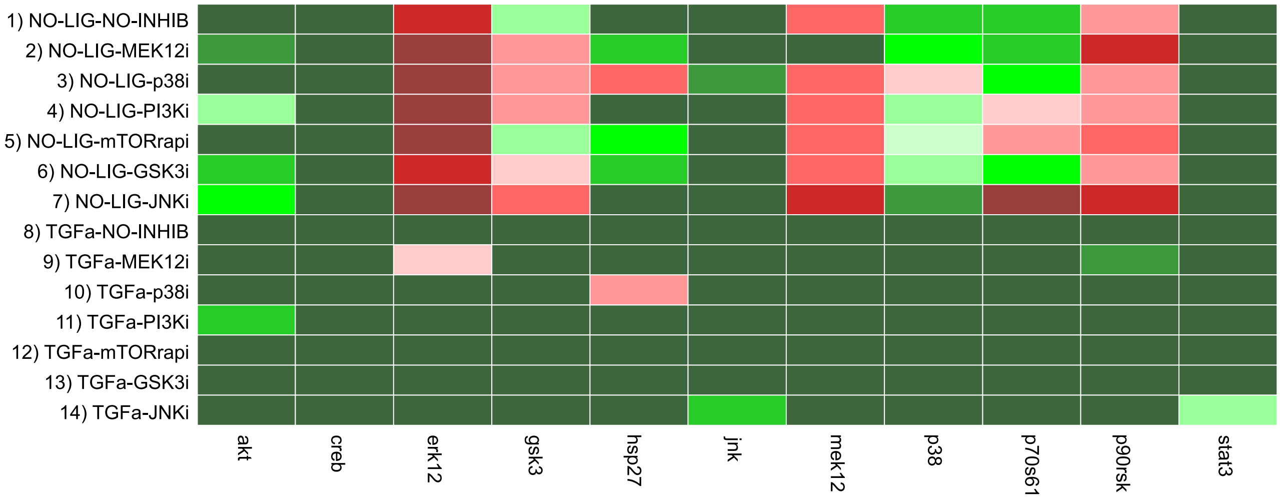

2.1c)

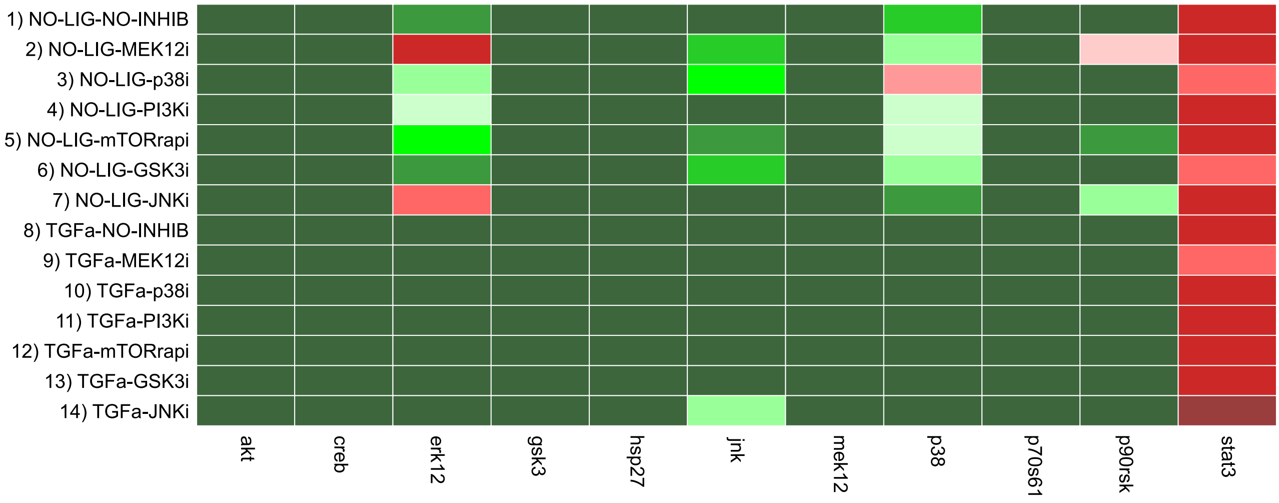
1) NO-LIG-NO-INHIB
2) NO-LIG-MEK12i
3) NO-LIG-p38i
4) NO-LIG-PI3Ki
5) NO-LIG-mTORrapi
6) NO-LIG-GSK3i
7) NO-LIG-JNKi
8) TGFa-NO-INHIB
9) TGFa-MEK12i
10) TGFa-p38i
11) TGFa-PI3Ki
12) TGFa-mTORrapi
13) TGFa-GSK3i
14) TGFa-JNKi
akt
creb
erk12
gsk3
hsp27
jnk
mek12
p38
p70s61
p90rsk
stat3

2.2a)

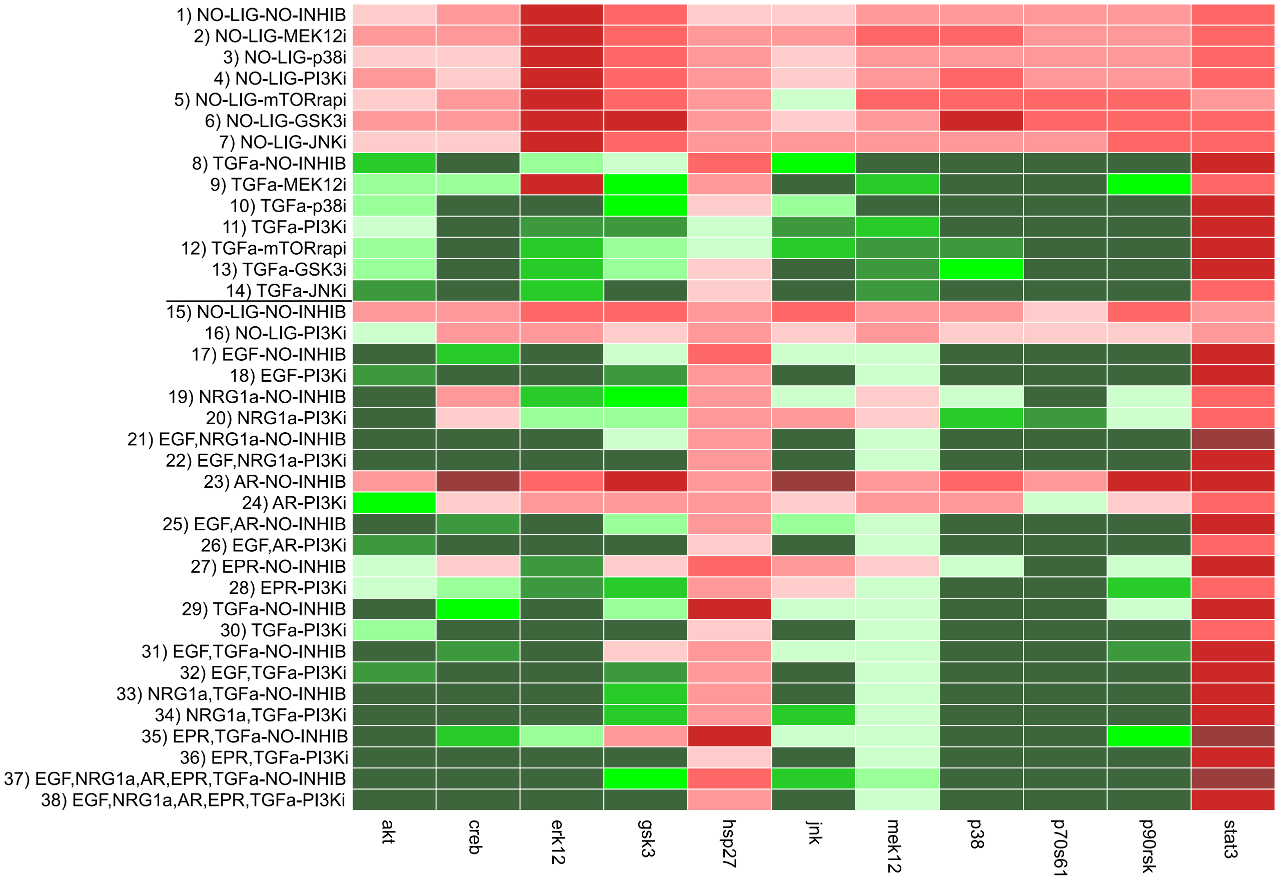
1) NO-LIG-NO-INHIB
2) NO-LIG-MEK12i
3) NO-LIG-p38i
4) NO-LIG-PI3Ki
5) NO-LIG-mTORrapi
6) NO-LIG-GSK3i
7) NO-LIG-JNKi
8) TGFa-NO-INHIB
9) TGFa-MEK12i
10) TGFa-p38i
11) TGFa-PI3Ki
12) TGFa-mTORrapi
13) TGFa-GSK3i
14) TGFa-JNKi
15) NO-LIG-NO-INHIB
16) NO-LIG-PI3Ki
17) EGF-NO-INHIB
18) EGF-PI3Ki
19) NRG1a-NO-INHIB
20) NRG1a-PI3Ki
21) EGF,NRG1a-NO-INHIB
22) EGF,NRG1a-PI3Ki
23) AR-NO-INHIB
24) AR-PI3Ki
25) EGF,AR-NO-INHIB
26) EGF,AR-PI3Ki
27) EPR-NO-INHIB
28) EPR-PI3Ki
29) TGFa-NO-INHIB
30) TGFa-PI3Ki
31) EGF,TGFa-NO-INHIB
32) EGF,TGFa-PI3Ki
33) NRG1a,TGFa-NO-INHIB
34) NRG1a,TGFa-PI3Ki
35) EPR,TGFa-NO-INHIB
36) EPR,TGFa-PI3Ki
37) EGF,NRG1a,AR,EPR,TGFa-NO-INHIB
38) EGF,NRG1a,AR,EPR,TGFa-PI3Ki
akt
creb
erk12
gsk3
hsp27
jnk
mek12
p38
p70s61
p90rsk
stat3

2.2b)

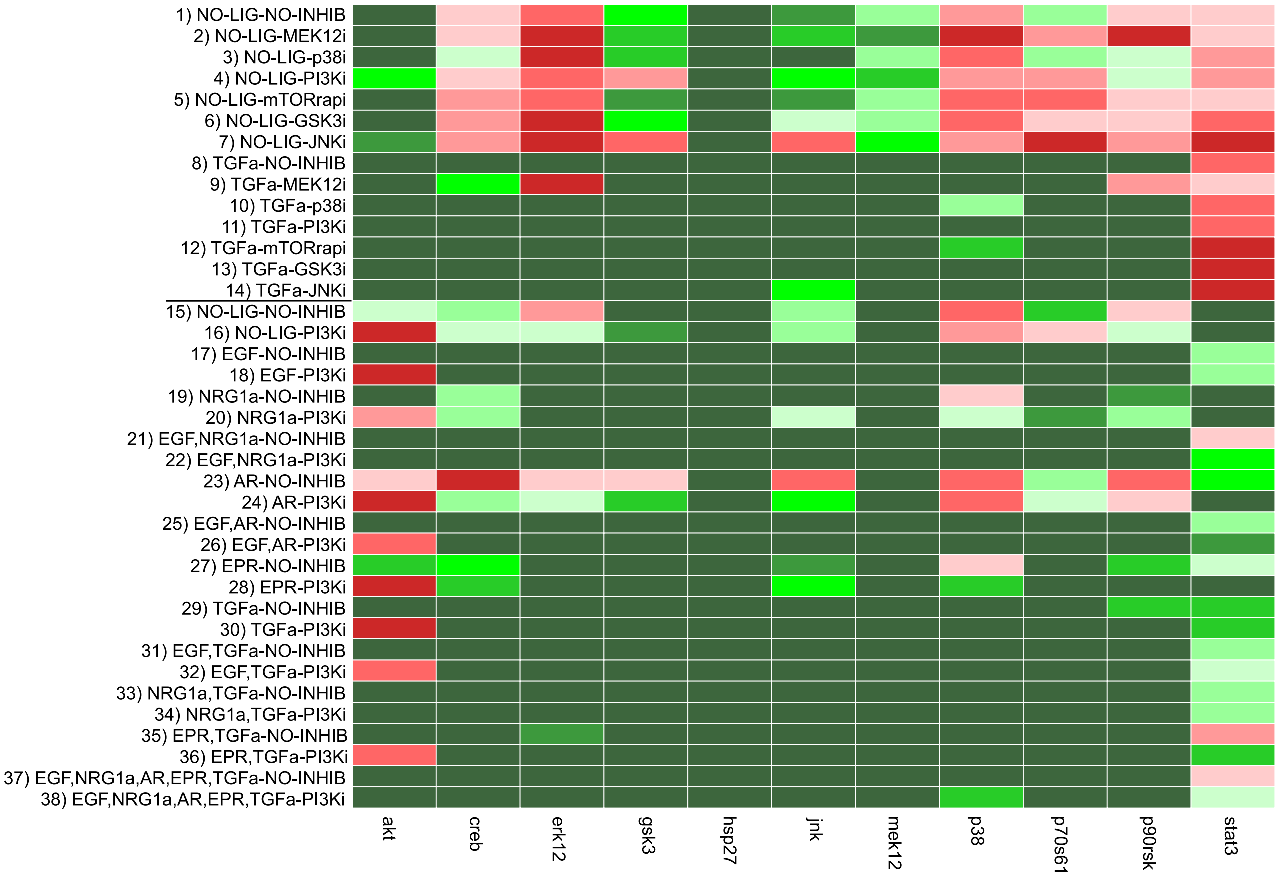
1) NO-LIG-NO-INHIB
2) NO-LIG-MEK12i
3) NO-LIG-p38i
4) NO-LIG-PI3Ki
5) NO-LIG-mTORrapi
6) NO-LIG-GSK3i
7) NO-LIG-JNKi
8) TGFa-NO-INHIB
9) TGFa-MEK12i
10) TGFa-p38i
11) TGFa-PI3Ki
12) TGFa-mTORrapi
13) TGFa-GSK3i
14) TGFa-JNKi
15) NO-LIG-NO-INHIB
16) NO-LIG-PI3Ki
17) EGF-NO-INHIB
18) EGF-PI3Ki
19) NRG1a-NO-INHIB
20) NRG1a-PI3Ki
21) EGF,NRG1a-NO-INHIB
22) EGF,NRG1a-PI3Ki
23) AR-NO-INHIB
24) AR-PI3Ki
25) EGF,AR-NO-INHIB
26) EGF,AR-PI3Ki
27) EPR-NO-INHIB
28) EPR-PI3Ki
29) TGFa-NO-INHIB
30) TGFa-PI3Ki
31) EGF,TGFa-NO-INHIB
32) EGF,TGFa-PI3Ki
33) NRG1a,TGFa-NO-INHIB
34) NRG1a,TGFa-PI3Ki
35) EPR,TGFa-NO-INHIB
36) EPR,TGFa-PI3Ki
37) EGF,NRG1a,AR,EPR,TGFa-NO-INHIB
38) EGF,NRG1a,AR,EPR,TGFa-PI3Ki
akt
creb
erk12
gsk3
hsp27
jnk
mek12
p38
p70s61
p90rsk
stat3

2.2c)

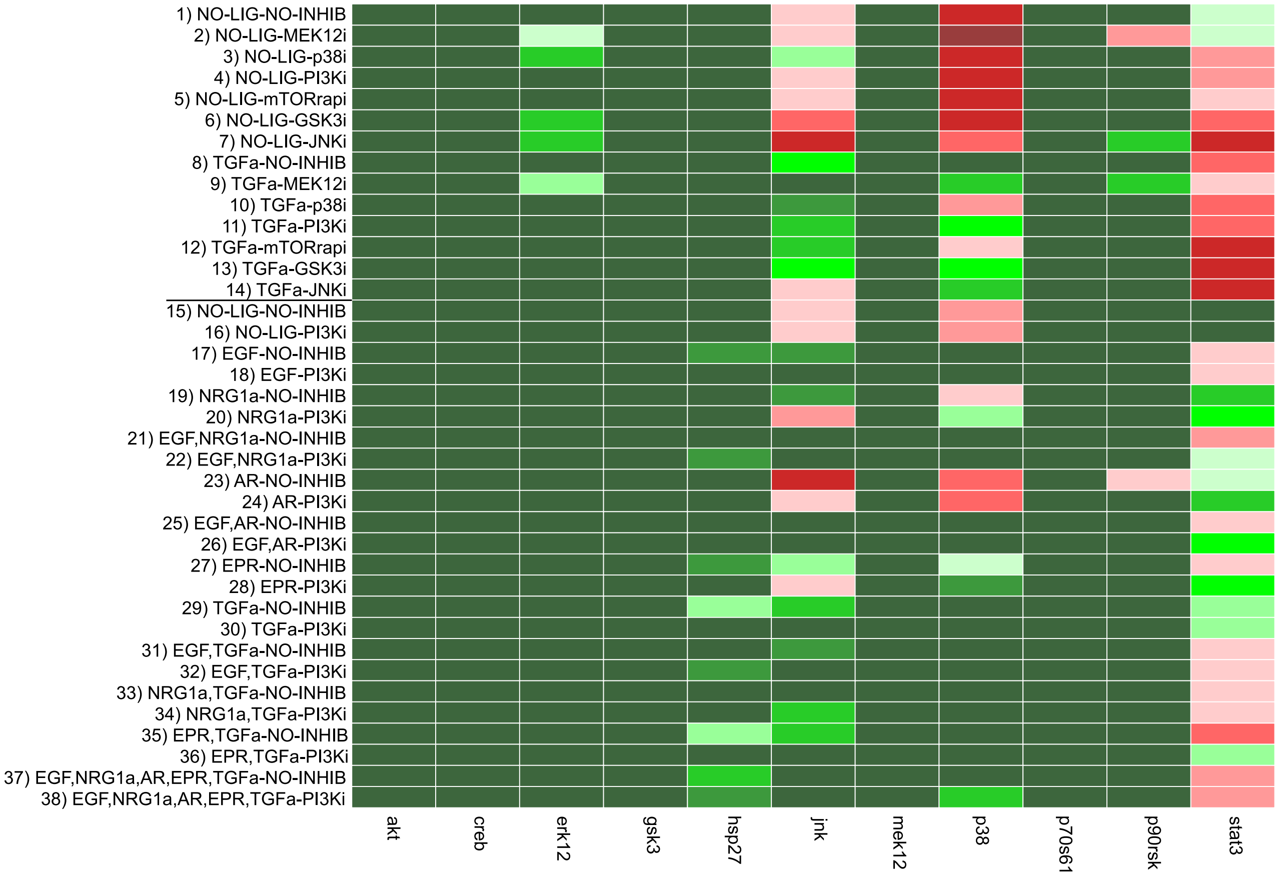
1) NO-LIG-NO-INHIB
2) NO-LIG-MEK12i
3) NO-LIG-p38i
4) NO-LIG-PI3Ki
5) NO-LIG-mTORrapi
6) NO-LIG-GSK3i
7) NO-LIG-JNKi
8) TGFa-NO-INHIB
9) TGFa-MEK12i
10) TGFa-p38i
11) TGFa-PI3Ki
12) TGFa-mTORrapi
13) TGFa-GSK3i
14) TGFa-JNKi
15) NO-LIG-NO-INHIB
16) NO-LIG-PI3Ki
17) EGF-NO-INHIB
18) EGF-PI3Ki
19) NRG1a-NO-INHIB
20) NRG1a-PI3Ki
21) EGF,NRG1a-NO-INHIB
22) EGF,NRG1a-PI3Ki
23) AR-NO-INHIB
24) AR-PI3Ki
25) EGF,AR-NO-INHIB
26) EGF,AR-PI3Ki
27) EPR-NO-INHIB
28) EPR-PI3Ki
29) TGFa-NO-INHIB
30) TGFa-PI3Ki
31) EGF,TGFa-NO-INHIB
32) EGF,TGFa-PI3Ki
33) NRG1a,TGFa-NO-INHIB
34) NRG1a,TGFa-PI3Ki
35) EPR,TGFa-NO-INHIB
36) EPR,TGFa-PI3Ki
37) EGF,NRG1a,AR,EPR,TGFa-NO-INHIB
38) EGF,NRG1a,AR,EPR,TGFa-PI3Ki
akt
creb
erk12
gsk3
hsp27
jnk
mek12
p38
p70s61
p90rsk
stat3
