# Supplementary material for: The Logic of EGFR/ErbB Signaling: Theoretical Properties and Analysis of High-Throughput Data
Source: PLoS Comput Biol. 2009 Aug 7;5(8):e1000438. doi: 10.1371/journal.pcbi.1000438 (PMC2710522; doi:10.1371/journal.pcbi.1000438)

Figure S3. Comparison of the discretized data with predictions from model M2.
A Primary human hepatocytes (data from Alexopoulos *et al*, in preparation).
B HepG2 cells (the horizontal line separates the the first (top) from the second (bottom) dataset for HepG2 cells; see also text).
Each row represents one treatment and the readouts are shown in the columns. Light green: predicted correctly, “on”; dark green: predicted correctly, “off”; light red: predicted “on”, measured “off”; dark red: predicted “off”, measured “on”; yellow: state cannot be determined in logical steady state analysis; black: data points where the measured species is inhibited are not considered.

A

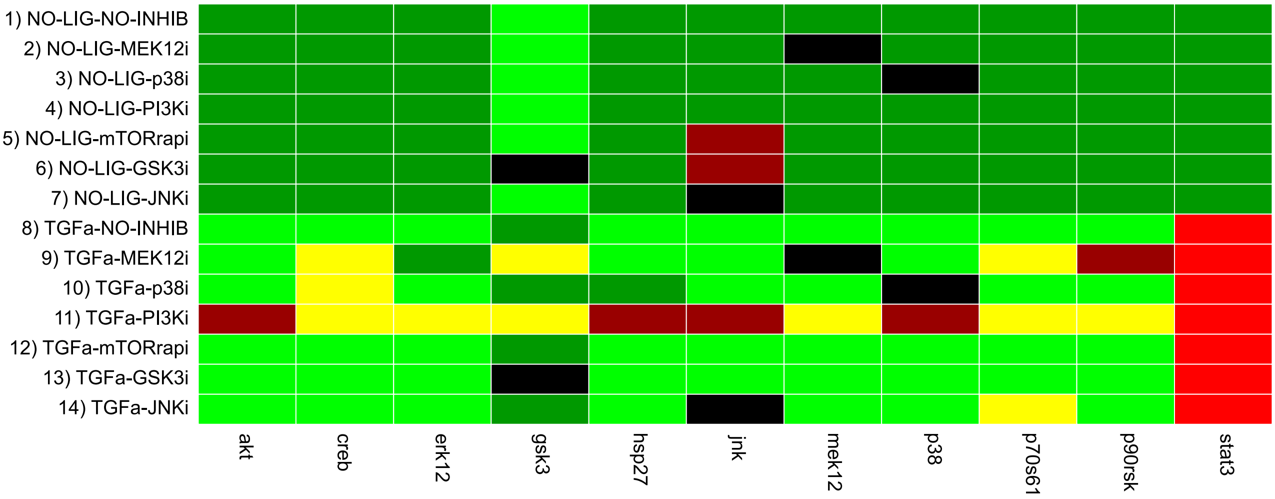

B

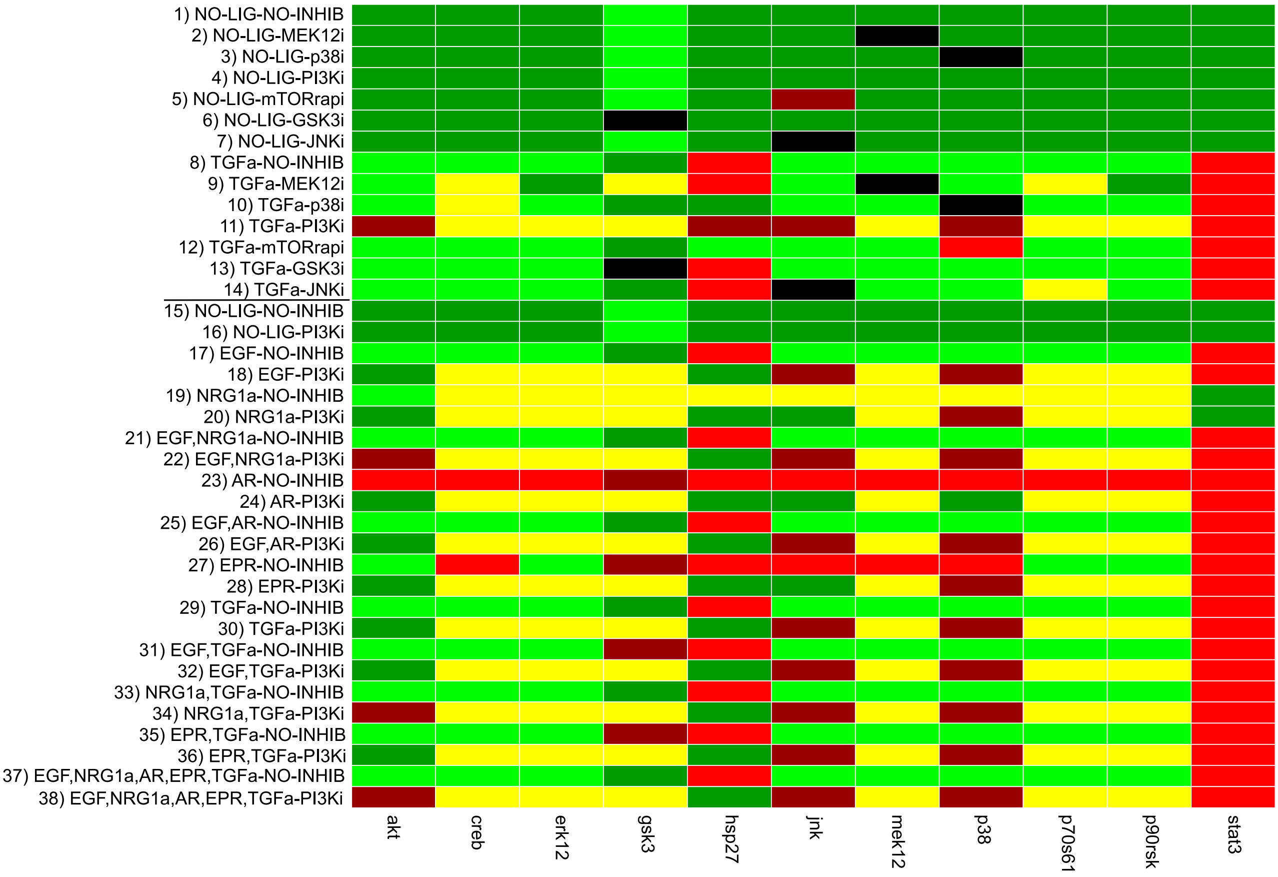

Supplement: Figure S3 — Comparison of the discretized data with predictions from model M2. A Primary human hepatocytes (data from Alexopoulos et al, in preparation). B HepG2 cells (the horizontal line separates the the first (top) from the second (bottom) dataset for HepG2 cells; see also text). Each row represents one treatment and the readouts are shown in the columns. Light green: predicted correctly, “on”; dark green: predicted correctly, “off”; light red: predicted “on”, measured “off”; dark red: predicted “off”, measured “on”; yellow: state cannot be determined in logical steady state analysis; black: data points where the measured species is inhibited are not considered. (0.21 MB PDF) [file pcbi.1000438.s003.pdf]
