# Supplementary material for: The Logic of EGFR/ErbB Signaling: Theoretical Properties and Analysis of High-Throughput Data
Source: PLoS Comput Biol. 2009 Aug 7;5(8):e1000438. doi: 10.1371/journal.pcbi.1000438 (PMC2710522; doi:10.1371/journal.pcbi.1000438)
Supplement: Figure S4 — Data plots generated with DataRail. Shown are the phosphorylation states of the proteins after 0, 30 and 180 minutes. Green: significant activation after 30 minutes (according to the chosen parameters); gray: no significant activation (cf. also Saez-Rodriguez et al, 2008). A Primary human hepatocytes (data obtained from Alexopoulos et al (in preparation)) B HepG2 cells, first set of experiments (data obtained from Alexopoulos et al (in preparation)) C HepG2 cells, second set of experiments. (0.28 MB PDF) [file pcbi.1000438.s004.pdf]

Figure S4. Data plots generated with *DataRail*.
Shown are the phosphorylation states of the proteins after 0, 30 and 180 minutes.
Green: significant activation after 30 minutes (according to the chosen parameters);
gray: no significant activation (cf. also Saez-Rodriguez *et al*, 2008).
A Primary human hepatocytes (data obtained from Alexopoulos *et al* (in preparation))
B HepG2 cells, first set of experiments (data obtained from Alexopoulos *et al* (in preparation))
C HepG2 cells, second set of experiments

A

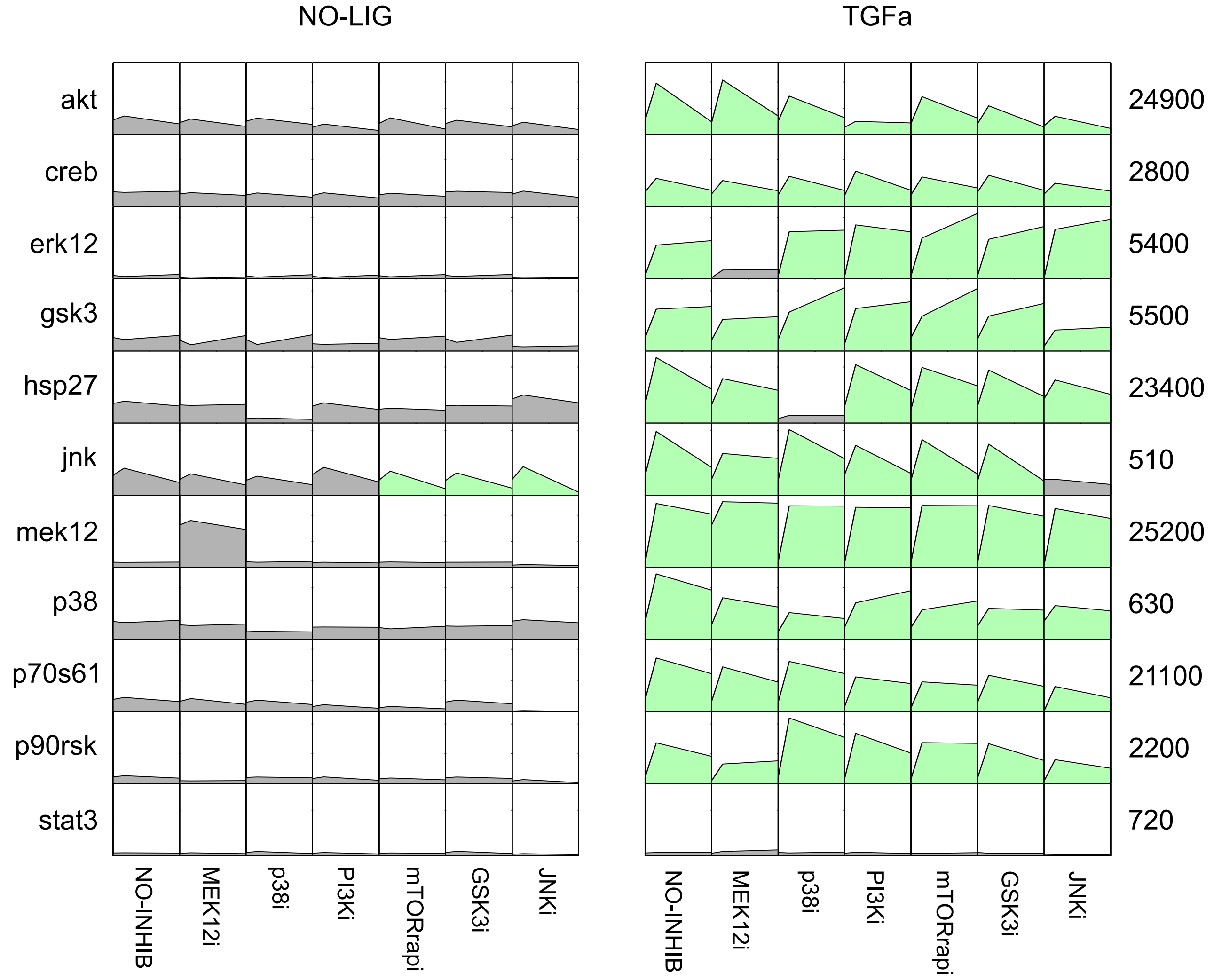

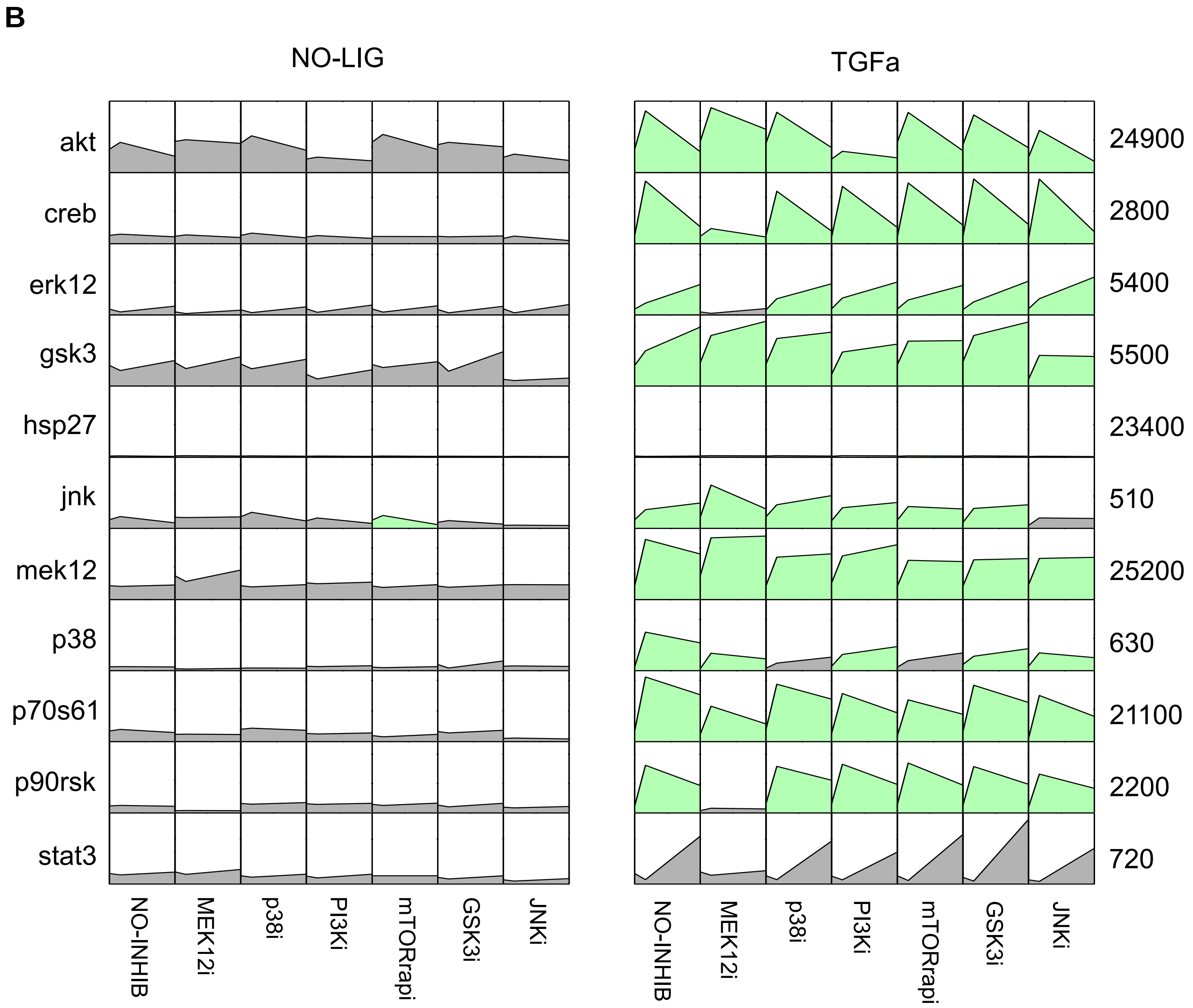
B
NO-LIG
TGFa
akt
creb
erk12
gsk3
hsp27
jnk
mek12
p38
p70s61
p90rsk
stat3
24900
2800
5400
5500
23400
510
25200
630
21100
2200
720
NO-INHIB
MEK12i
p38i
PI3Ki
mTORrapi
GSK3i
JNKi

C

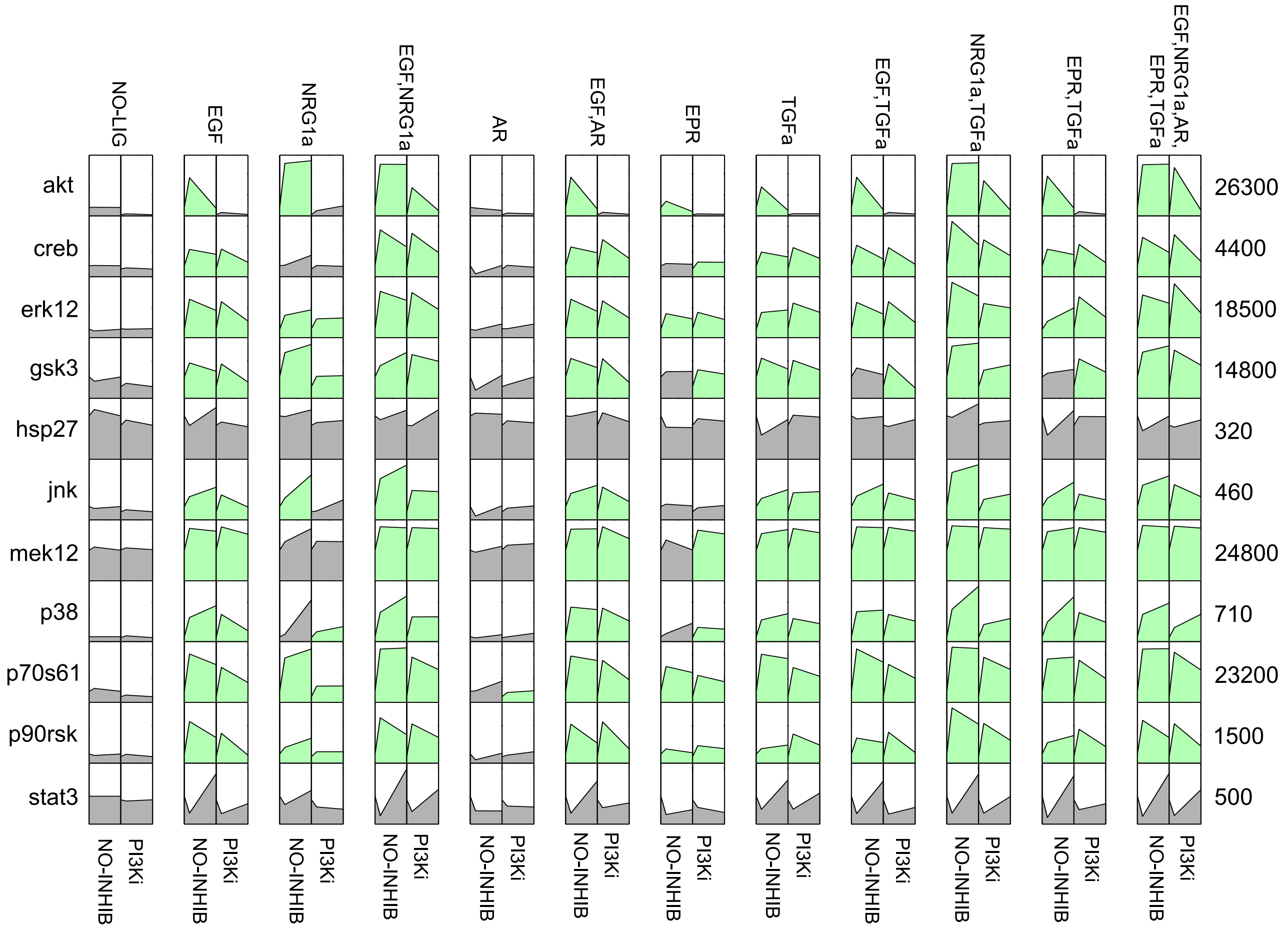
NO-LIG
EGF
NRG1a
EGF,NRG1a
AR
EGF,AR
EPR
TGFa
EGF,TGFa
NRG1a,TGFa
EPR,TGFa
EGF,NRG1a,AR, EPR,TGFa
akt
creb
erk12
gsk3
hsp27
jnk
mek12
p38
p70s61
p90rsk
stat3
26300
4400
18500
14800
320
460
24800
710
23200
1500
500
NO-INHIB
PI3Ki
