# Supplementary material for: The Logic of EGFR/ErbB Signaling: Theoretical Properties and Analysis of High-Throughput Data
Source: PLoS Comput Biol. 2009 Aug 7;5(8):e1000438. doi: 10.1371/journal.pcbi.1000438 (PMC2710522; doi:10.1371/journal.pcbi.1000438)
Supplement: Table S1 — Logical EGFR/ErbB model: list of species and interactions. (0.16 MB PDF) [file pcbi.1000438.s005.pdf]

Table S1.1. List of species in the logical EGFR/ErbB model.

Nr	model name	full name	documentation
1	actin_reorg	actin reorganization	symbolizes actin reorganizing effects initiated by LIMK1
2	akt		also known as PKB (protein kinase B)
3	aktd		dummy species for akt
4	ap1	activating protein 1	Collective term referring to dimeric transcription factors composed of jun, fos or ATF subunits, here: heterodimer composed of c-Jun and c-Fos.
5	pro_apoptotic		symbolizes the pro-apoptotic effect of BAD
6	ar	amphiregulin	ligand that binds specifically ErbB1
7	bad	BCL2-antagonist of cell death	phosphorylated (the unphosphorylated form promotes apoptosis)
8	bir	biregulin	synthetic neuregulin/egf chimera
9	btc	betacellulin	ligand with dual specificity, binds ErbB1 and ErbB4
10	ca	calcium	cytosolic Ca^{2+} -ions
11	ccbl	Cas-Br-M (murine) ecotropic retroviral transforming sequence	
12	cfos	v-fos FBJ murine osteosarcoma viral oncogene	
13	cjun	v-jun sarcoma virus 17 oncogene homolog	
14	cmyc	v-myc myelocytomatosis viral oncogene homolog	
15	creb	CRE (cAMP-responsive element) -binding protein	
16	csrc	v-src sarcoma (Schmidt-Ruppin A2) viral oncogene homolog (avian)	
17	dag	diacylglycerol	
18	egf	epidermal growth factor	ligand that binds specifically ErbB1
19	elk1	ELK1, member of ETS oncogene family	
20	endocyt_degrad		symbolizes endocytosis/degradation of the receptors
21	epr	epiregulin	ligand with dual specificity, binds ErbB1 and ErbB4
22	eps8r	epidermal growth factor receptor pathway substrate 8	reservoir of Eps8
23	erbb1	epidermal growth factor receptor	
24	erbb11		homodimer composed of two ErbB1-receptors
25	erbb12		heterodimer composed of ErbB1-and ErbB2-receptor
26	erbb13		heterodimer composed of ErbB1-and ErbB3-receptor
27	erbb14		heterodimer composed of ErbB1-and ErbB4-receptor

28	erbb2	v-erb-b2 erythroblastic leukemia viral oncogene homolog 2, neuro/glioblastoma derived oncogene homolog (avian)	member of the ErbB familiy, no ligand, preferred heterodimerization partner of the other ErbBs; also known as HER2
29	erbb23		heterodimer composed of ErbB2-and ErbB3-receptor
30	erbb24		heterodimer composed of ErbB2-and ErbB4-receptor
31	erbb3	v-erb-b2 erythroblastic leukemia viral oncogene homolog 3 (avian)	member of the ErbB family, kinase-defective; also known as HER3
32	erbb34		heterodimer composed of ErbB3-and ErbB4-receptor
33	erbb4	v-erb-a erythroblastic leukemia viral oncogene homolog 4 (avian)	member of the ErbB family;<br>also known as HER4
34	erbb44		homodimer composed of two ErbB4-receptors
35	erk12	mitogen activated protein kinase 1 or 3	Since both ERK1 and ERK2 catalyze the same reactions, we do not distinguish between them
36	gab1	Grb2-associated binding protein 1	
37	grb2	growth factor receptor bound protein 2	
38	gsk3	glycogen synthase kinase 3 beta	
39	hbegf	heparin-binding EGF	ligand with dual specificity, binds ErbB1 and ErbB4
40	hsp27	heat shock protein 27	
41	ip3	Inositol-1,4,5-triphosphat	
42	jnk	mitogen-activated protein kinase 8	
43	limk1	LIM domain kinase 1	
44	mek12	mitogen-activated protein kinase kinase 1 or 2	Since both MEK1 and MEK2 catalyze the same reactions, we do not distinguish between them;<br>also known as MKK1/MKK2
45	mekk1	mitogen-activated protein kinase kinase kinase 1	
46	mekk4	mitogen-activated protein kinase kinase kinase 4	
47	mk2	mitogen-activated protein kinase-activated protein kinase 2	
48	mkk3	mitogen-activated protein kinase kinase 3	
49	mkk4	mitogen-activated protein kinase kinase 4	
50	mkk6	mitogen-activated protein kinase kinase 6	
51	mkk7	mitogen-activated protein kinase kinase 7	
52	mkp	dual specificity phosphatase 1	

53	mlk3	mitogen-activated protein kinase kinase kinase 11	
54	mtorr	mammalian target of rapamycin	reservoir of mtor
55	mtor_rap		mTOR-raptor complex
56	mtor_ric		mTOR-rictor complex
57	nck	NCK adaptor protein 1	
58	nrg1a	neuregulin-1α	ligand with dual specificity, binds ErbB3 and ErbB4
59	nrg1b	neuregulin-1β	ligand with dual specificity, binds ErbB3 and ErbB4
60	nrg2a	neuregulin-2α	ligand with dual specificity, binds ErbB3 and ErbB4
61	nrg2b	neuregulin-2β	ligand with dual specificity, binds ErbB3 and ErbB4
62	nrg3	neuregulin 3	ligand that binds specifically ErbB4
63	nrg4	neuregulin 4	ligand that binds specifically ErbB4
64	nucerk12		phosphorylated dimer of ERK1/2, located in the nucleus
65	p38	mitogen-activated protein kinase 14	
66	p70s6_1	ribosomal protein S6 kinase, 70kDa, polypeptide 1	p70s6 phosphorylated at autoinhibitory sites
67	p70s6_2		p70s6 phosphorylated at autoinhibitory sites and at the catalytic sites T389 and S229
68	p90rsk	ribosomal protein S6 kinase, 90kDa, polypeptide 1	
69	p90rskerk12d		combined dummy species for p90Rsk and ERK1/2
70	pak1	p21/Cdc42/Rac1-activated kinase	
71	pdk1	3-phosphoinositide dependent protein kinase-1	
72	pi34p2	Phosphatidylinositol-3,4-biphosphat	Membranlipid
73	pi3k	phosphatidylinositol-3-kinase	
74	pi3kr		reservoir of PI3K
75	pip3	Phosphatidylinositol-3,4,5-triphosphat	Membranlipid
76	pkc	protein kinase C	
77	plcg	phospholipase c gamma	
78	pp2a	protein phosphatase 2A	
79	pp2b	protein phosphatase 2B	also known as calcineurin
80	pten	phosphatase and tensin homolog	
81	ptend		dummy species for PTEN
82	rab5a	Ras-associated protein	
83	rac_cdc42		small GTPase Rac or Cdc42
84	raf1	v-raf-1 murine leukemia viral oncogene homolog 1	
85	ras	rat sarcoma viral oncogene homolog	
86	rasgap	RAS p21 protein activator (GTPase activating protein)1	
87	rheb	Ras homolog enriched in brain	
88	rin1	RAS and RAB interactor 1	guanine nucleotide exchange factor, specific for Rab5 proteins

89	rntre	related to the N terminus of tre	GTPase activating protein, bound to Eps8 and ErbB1-dimers
90	shc	src homology 2 domain containing transforming protein 1	
91	ship2	inositol polyphosphate phosphatase-like 1	
92	ship2d		dummy species for SHIP2
93	shp1	protein tyrosine phosphatase, non-receptor type 6	
94	shp1d		dummy species for SHP1
95	shp2	protein tyrosine phosphatase, non-receptor type 11	
96	sos1	son of sevenless homolog 1	
97	sos1r		reservoir of SOS1
98	sos1_eps8_e3b1		complex of SOS1, Eps8 and E3b1
99	stat1	signal transducer and activator of transcription 1	
100	stat3	signal transducer and activator of transcription 3	
101	stat5	signal transducer and activator of transcription 5	
102	tgfa	transforming growth factor alpha	ligand that binds specifically ErbB1
103	tsc1_tsc2	tuberous sclerosis complex 1 and 2	
104	vav2	vav2 oncogene	

Table S1.2. List of interactions in the logical EGFR/ErbB model.

Notation:

→ A	species A is an input to the model
A →	species A is an output of the model
A → B	species A activates species B
A · B → C	species A AND B activate C (and both A and B are necessary for activation)
A · !B → C	species C is activated when A AND NOT B are present

Activation of the ErbB-dimers

The four ErbB receptors form different homo- and heterodimers. Binding of a ligand leads to autophosphorylation of tyrosine residues that provide docking sites for proteins with SH2 or PTB domains. As ErbB2 does not bind to ligands of the EGF family (Citri & Yarden, 2006), ErbB2 can only be activated in heterodimers. However, ErbB2 is the preferred heterodimerization partner of the other ErbBs and therefore the other heterodimers 13, 14 and 34 are only formed in absence of ErbB2. An exception are amphiregulin and HB-EGF activated dimers: AR activates ErbB3, but not ErbB2 (Beerli & Hynes, 1996), so we assume that AR activates ErbB13 dimers also in presence of ErbB2. ErbB3 is kinase-defective, thus ErbB3-homodimers are inactive (Olayioye *et al*, 2000; Citri & Yarden, 2006). According to Landau & Ben-Tal (2008), only one of the receptors in a dimer is phosphorylated. In ErbB1/ErbB2 dimers, only ErbB2 becomes phosphorylated (Landau & Ben-Tal, 2008). As ErbB3 is kinase-defective, ErbB3 is the phosphorylated partner in ErbB3-heterodimers (Landau & Ben-Tal, 2008). We could not find any information on the phosphorylation of ErbB1/ErbB4 and ErbB2/ErbB4 dimers. Therefore, we assume that proteins that can only bind to ErbB1 are activated through ErbB1 homodimers; proteins that can bind to ErbB2 are activated through ErbB1/ErbB2 dimers; proteins that can bind to ErbB3 are activated through all possible ErbB3-dimers (13, 23, 34) and proteins that can bind to ErbB4 are activated through ErbB4 homodimers.

Nr	interaction	time	value	documentation
1	erbb11 → shp1	1		SHP1 binds to ErbB1 at phosphorylated Y1173 (Keilhack *et al*, 1998).
2	shp1 → shp1d	2		SHP1 dephosphorylates the ErbB1 dimers (negative feedback; see e.g. reaction 8). We assume this dephosphorylation to be a late event and therefore have to include a dummy species.
3	→ erbb1	1	1	
4	→ erbb2	1	1	
5	→ erbb3	1	1	
6	→ erbb4	1	1	
7	→ ar	1	0	
8	ar · !shp1d · erbb1 → erbb11	1		Amphiregulin binds ErbB1-homodimers and ErbB13 – however, the affinity of AR towards ErbB1 is significantly lower than the affinity of EGF (Beerli & Hynes, 1996). Reaction 9 is not included in Oda *et al* (2005).
9	ar · !shp1d · erbb1 · erbb3 → erbb13	1		
10	→ bir	1	0	
11	bir · !shp1d · erbb1 → erbb11	1		Biregulin activates the following ErbB dimers: 11, 12, 23, 24, 44 (Jones *et al*, 1999). Since in Jones *et al* (1999) the dimers 13, 14 and 34 are not analyzed and this is the only source about binding affinities for biregulin we found, we cannot rule out the possibility that 13, 14 and 34 are also activated. As biregulin is an artificial ligand, one could think about not considering it in the model.
12	bir · !shp1d · erbb1 · erbb2 → erbb12	1		
13	bir · erbb2 · erbb3 → erbb23	1		
14	bir · erbb2 · erbb4 → erbb24	1		
15	bir · erbb4 → erbb44	1		

16	→ btc	1	0	
17	btc · !shp1d · erbb1 → erbb11	1		Betacellulin activates the following ErbB-dimers: 11, 12, 24, 44 (Jones *et al*, 1999). Additionally, it activates 13 (Alroy & Yarden, 1997) (not part of Oda *et al* (2005)). In Alroy & Yarden (1997) activation of 14 was detected, whereas this is not reported in Graus-Porta *et al* (1997). Therefore we decided not to include activation of 14 so far. In Wang *et al* (1998) and Graus-Porta *et al* (1997) activation of 23 is reported, what is contradictory to Jones *et al* (1999) and not mentioned in Alroy & Yarden (1997). However, it is in accordance with the findings of Beerli & Hynes (1996) that BTC activates ErbB3 when all ErbB receptors are present and is thus included in the model.
18	btc · !shp1d · erbb1 · erbb2 → erbb12	1		
19	btc · !shp1d · erbb1 · !erbb2 · erbb3 → erbb13	1		
20	btc · erbb2 · erbb3 → erbb23	1		
21	btc · erbb2 · erbb4 → erbb24	1		
22	btc · erbb4 → erbb44	1		
23	→ egf	1	0	
24	egf · !shp1d · erbb1 → erbb11	1		EGF activates the following ErbB-dimers: 11, 12, 24 (Jones *et al*, 1999). In absence of ErbB2, also 13 and 14 can be activated (Graus-Porta *et al*, 1997; Olayioye *et al*, 1998). Activation of 13 and 14 is not included in Oda *et al* (2005). Furthermore, in Wang *et al* (1998) and Graus-Porta *et al* (1997) activation of 23 is mentioned. We decided not to consider this in the model so far for two reasons: First, in Jones *et al* (1999) 23 dimers were studied, but no measurable binding of EGF was detected. Second, in Shelly *et al* (1998) it is stated that this activation occurs only at very high ligand concentrations.
25	egf · !shp1d · erbb1 · erbb2 → erbb12	1		
26	egf · !shp1d · erbb1 · !erbb2 · erbb3 → erbb13	1		
27	egf · !shp1d · erbb1 · !erbb2 · erbb4 → erbb14	1		
28	egf · erbb2 · erbb4 → erbb24	1		
29	!endocyt_degrad → erbb11	2		ErbB1-homodimers activated through EGF are endocytosed and subsequently degraded – in contrast to TGFα -bound receptors (Lenferink *et al*, 1998) and the other ErbB-dimers. As we have not included the detailed endocytosis mechanism in the model so far, we do not distinguish between EGF-bound and TGFα –bound receptors at the moment. Internalized receptors are still capable of activating signaling pathways (Citri & Yarden, 2006), so we decided to exclude this reaction in the logical analysis.
30	→ epr	1	0	
31	epr · !shp1d · erbb1 → erbb11	1		Epiregulin activates the following ErbB-dimers: 11, 12, 23, 24 (Jones *et al*, 1999). In Shelly *et al* (1998) additionally activation of 13, 14 (high) and 34, 44 (low) is mentioned (none of these part of the map of Oda *et al* (2005)). We included activation of 13 and 14 in the model, because these heterodimers are not part of the analysis in Jones *et al* (1999) and thus the results in Jones *et al* (1999) and Shelly *et al* (1998) are not contradictory. Activation of 44 was not considered, because this is contradictory to Jones *et al* (1999) and furthermore was mentioned as low in Shelly *et al* (1998). We think of including the interaction with 34, but did not realize it so far, because the activation is also mentioned as low.
32	epr · !shp1d · erbb1 · erbb2 → erbb12	1		
33	epr · !shp1d · erbb1 · erbb3 · !erbb2 → erbb13	1		
34	epr · !shp1d · erbb1 · erbb4 · !erbb2 → erbb14	1		
35	epr · erbb2 · erbb3 → erbb23	1		
36	epr · erbb2 · erbb4 → erbb24	1		

37	→ hbegf	1	0	
38	hbegf · !shp1d · erbb1 → erbb11	1		HB-EGF activates the following ErbB-dimers: 11, 12, 24 (Jones *et al*, 1999). In Beerli & Hynes (1996) activation of ErbB3 in response to HB-EGF is stated – in presence of ErbB2. One possibility could be that HB-EGF activates ErbB13 dimers even in presence of ErbB2. Alternatively, HB-EGF might activate ErbB23 dimers, contradictory to the findings in Jones *et al* (1999).
39	hbegf · !shp1d · erbb1 · erbb2 → erbb12	1		
40	hbegf · erbb2 · erbb4 → erbb24	1		
41	→ nrg1a	1	0	
42	nrg1a · !shp1d · erbb1 · erbb3 · !erbb2 → erbb13	1		NRG-1α activates the following ErbB dimers: 23, 24, 44. Furthermore (not mentioned in Oda *et al* (2005)), it activates 13 (Olayioye *et al*, 1998; Graus-Porta *et al*, 1997; Alroy & Yarden, 1997), 14 (Pinkas-Kramarski *et al*, 1998; (Olayioye *et al*, 1998; Graus-Porta *et al*, 1997; Alroy & Yarden, 1997) and 34 (Pinkas-Kramarski *et al*, 1998; Alroy & Yarden, 1997).
43	nrg1a · !shp1d · erbb1 · erbb4 · !erbb2 → erbb14	1		
44	nrg1a · erbb2 · erbb3 → erbb23	1		
45	nrg1a · erbb2 · erbb4 → erbb24	1		
46	nrg1a · erbb4 → erbb44	1		
47	nrg1a · erbb3 · erbb4 · !erbb2 → erbb34	1		
48	→ nrg1b	1	0	
49	nrg1b · !shp1d · erbb1 · erbb3 · !erbb2 → erbb13	1		NRG-1β activates the following ErbB dimers: 23, 24, 44 (Jones *et al*, 1999). In accordance with Pinkas-Kramarski *et al* (1998) and Alroy & Yarden (1997) also 13, 14 and 34 can be activated (not depicted in Oda *et al* (2005)).
50	nrg1b · !shp1d · erbb1 · erbb4 · !erbb2 → erbb14	1		
51	nrg1b · erbb2 · erbb3 → erbb23	1		
52	nrg1b · erbb2 · erbb4 → erbb24	1		
53	nrg1b · erbb4 → erbb44	1		
54	nrg1b · erbb3 · erbb4 · !erbb2 → erbb34	1		
55	→ nrg2a	1	0	
56	nrg2a · !shp1d · erbb1 · erbb3 · !erbb2 → erbb13	1		NRG-2α activates the ErbB dimer 24 (Jones *et al*, 1999). Additionally, 13, 14 and 34 are activated (Pinkas-Kramarski *et al*, 1998) (not included in Oda *et al* (2005)). In Pinkas-Kramarski *et al* (1998), activation of 11, 12, 23 and 44 is also reported. We decided not to include this in the model so far, since these interactions are in contradiction to Jones *et al* (1999).
57	nrg2a · !shp1d · erbb1 · erbb4 · !erbb2 → erbb14	1		
58	nrg2a · erbb2 · erbb4→ erbb24	1		
59	nrg2a · erbb3 · erbb4 · !erbb2 → erbb34	1		
60	→ nrg2b	1	0	
61	nrg2b · !shp1d · erbb1 · erbb4 · !erbb2 → erbb14	1		In accordance with Jones *et al* (1999), NRG-2β activates the following ErbB dimers: 23, 24, 44. Furthermore, activation of 14 and 34 is reported in Pinkas-Kramarski *et al* (1998) (not depicted in Oda *et al* (2005)).
62	nrg2b · erbb2 · erbb3 → erbb23	1		
63	nrg2b · erbb2 · erbb4 → erbb24	1		
64	nrg2b · erbb3 · erbb4 · !erbb2 → erbb34	1		
65	nrg2b · erbb4 → erbb44	1		

66	→ nrg3	1	0	
67	nrg3 · erbb2 · erbb4 → erbb24	1		NRG3 activates the following ErbB dimers: 24, 44 (Jones *et al*, 1999). However, in Jones *et al* (1999) 13, 14 and 34 are not analyzed. Therefore and since we were not able to find another paper dealing with binding specificities of NRG3, we cannot be sure that there is no interaction between NRG3 and these dimers.
68	nrg3 · erbb4 → erbb44	1		
69	→ nrg4	1	0	
70	nrg4 · !shp1d · erbb1 · erbb4 · !erbb2 → erbb14	1		NRG4 activates the following ErbB dimers: 14, 24, 44 (Harari *et al*, 1999). In Oda *et al* (2005) also activation of 34 is depicted. However, we could not find a source where this is described and thus decided not to include it in the model so far.
71	nrg4 · erbb2 · erbb4 → erbb24	1		
72	nrg4 · erbb4 → erbb44	1		
73	→ tgfa	1	0	
74	tgfa · !shp1d · erbb1 → erbb11	1		TGFα activates the following ErbB-dimers: 11, 12, 24 (Jones *et al*, 1999). In Alroy & Yarden (1997), activation of 13 and 14 (not part of the map of Oda *et al* (2005)) is mentioned.
75	tgfa · !shp1d · erbb1 · erbb2 → erbb12	1		
76	tgfa · !shp1d · erbb1 · !erbb2 · erbb3 → erbb13	1		
77	tgfa · !shp1d · erbb1 · !erbb2 · erbb4 → erbb14	1		
78	tgfa · erbb2 · erbb4 → erbb24	1		

**Activation of adaptor proteins**				
79	erbb11 → shc	1		Shc binds to all types of ErbB-receptors. On ErbB1, the binding sites are pY1148 (via PTB domain) and pY1173 (via PTB and SH2 domain) (Olayioye *et al*, 2000).
80	erbb12 → shc	1		
81	erbb13 → shc	1		
82	erbb14 → shc	1		
83	erbb23 → shc	1		
84	erbb24 → shc	1		
85	erbb34 → shc	1		
86	erbb44 → shc	1		
87	shc → grb2	1		Grb2 can bind to ErbB-dimers via Shc (Okabayashi *et al*, 1994) or directly via its SH2 domain. As there are binding sites for Grb2 on all ErbB receptors (Schulze *et al*, 2005), we assume that Grb2 can directly interact with all possible ErbB-dimers.
88	erbb11 → grb2	1		
89	erbb12 → grb2	1		
90	erbb13 → grb2	1		
91	erbb14 → grb2	1		
92	erbb23 → grb2	1		
93	erbb24 → grb2	1		
94	erbb34 → grb2	1		
95	erbb44 → grb2	1		
96	erbb11 → gab1	1		Gab1 can bind directly to ErbB1 receptors or via Grb2 and is phosphorylated on Y-residues by the receptor kinase (Rodrigues *et al*, 2000).
97	grb2 → gab1	1		

98	pip3 → gab1	2		PIP3 recruits Gab1 molecules to the EGFR and thus enhances the activity of Gab1 (Rodrigues *et al*, 2000). As we do not consider multilevel activation, we decided to exclude this positive feedback loop in the logical analysis. For the analysis of the interaction graph, we assume that the reaction is time delayed as it is part of the negative feedback loop from Ras via PI3K to RasGAP (see main text).
99	erbb11 → nck	1		Nck binds to EGFR (Li *et al*, 2001). In Schulze *et al* (2005), a Nck binding site on ErbB4 is reported.
100	erbb14 → nck	1		
101	erbb44 → nck	1		
**Activation of the G-Proteins ras and rac**				
102	→ sos1r	1	1	We consider two different pools of SOS1: activated through Grb2, leading to Ras-GEF activity (reaction 104) and in a complex with Eps8 and E3b1 (reaction 111), leading to Rac-GEF activity
103	p90rsk · erk12 → p90rskerk12d	2		Reaction that is introduced for modeling the time delay p90RSK and ERK1/2 phosphorylate and thus inhibit SOS1 with (see reaction 104).
104	sos1r · grb2 · !p90rskerk12d → sos1	1		SOS1 is bound to Grb2. Binding of Grb2 to EGFR, either directly or indirectly through Shc, leads to activation of SOS1. Serine/threonine phosphorylation of SOS1 by p90RSK or ERK1/2 causes dissociation of Grb2-SOS1 from Shc or from the phosphorylated receptor (Douville & Downward, 1997).
105	gab1 → shp2	1		Phosphorylated Gab1 recruits and activates SHP2 (Montagner *et al*, 2005).
106	gab1 · !shp2 → rasgap	1		RasGAP can bind tyrosine phosphorylation sites on Gab1. Gab1-bound SHP2 dephosphorylates these sites (Montagner *et al*, 2005). Note that this reaction is not part of the map of Oda *et al* (2005).
107	sos1 · !rasgap → ras	1		Interaction with SOS1 increases the rate of GDP/GTP exchange of Ras (and thus acts as activating GEF) (Li *et al*, 1993). The GTPase activating protein RasGAP leads to hydrolysis of Ras-bound GTP to GDP (Cox & Der, 2003).
108	erbb11 · pip3 → vav2	1		The SH2 domain of Vav2 binds to pY992 or pY1148 on ErbB1. The receptor phosphorylates Vav2 on Y142, Y159 and Y172. Both PIP3 and PI(3,4)P2 mediate the nucleotide exchange activity of Vav2 towards Rac and Cdc42 (Tamás *et al*, 2003). In Oda *et al* (2005), PI3K instead of PIP3/PI(3,4)P2 is depicted to influence the Rac-GEF activity of Vav2.
109	erbb11 · pi34p2 → vav2	1		
110	→ eps8r	1	1	Eps8 can form a complex with SOS1/E3b1 (reaction 111) or RN-tre (reaction 196).
111	sos1r · eps8r · pi3kr · pip3 → sos1eps8e3b1	1		SOS1, Eps8 and E3b1/Abi1 form a complex which is necessary for the Rac-GEF activity of SOS1. Furthermore, binding of the p85 subunit of PI3K to the complex is required for a basal Rac-GEF activity, which is increased by PIP3 (Innocenti *et al*, 2003). Note that this reaction differs from the description in the map of Oda *et al* (2005): There, only Ras-activated PI3K influences the GEF-activaty, whereas we assume the inactivated form to mediate this effect. Additionally, the influence of PIP3 is not considered in the map of Oda *et al* (2005).

112	vav2 → raccdc42	1		Both Vav2 and SOS1 (complexed with Eps8 and E3b1) act as GEF for Rac and Cdc42 (Innocenti *et al*, 2003; Tamás *et al*, 2003). As we did not find any indication in the literature for Vav2 and SOS1 activating Rac/Cdc42 cooperatively, we assume that both proteins can catalyze the GDP/GTP exchange independently of each other.
113	sos1eps8e3b1 → raccdc42	1		
**Activation of STATs**				
114	→ csrc	1	1	STAT1, 3 and 5 can be activated through ErbB1-homodimers whereas ErbB1 heterodimers do not seem to contribute to STAT activation. Neuregulin, which cannot activate ErbB1 dimers, induces activation of STAT5 through ErbB24. After ligand binding, Src is recruited to the activated receptor and phosphorylates receptor bound STATs on the consensus C-terminal Y-residue (Olayioye *et al*, 1999). It is not clear how Src is activated. Whereas in Sato *et al* (2002) activation of Src by Shc (in response to EGF) is reported that leads to phosphorylation of STAT, in Olayioye *et al* (1999) the activation of STAT after EGF stimulation was more rapid than activation of Src in response to EGF. Therefore we decided not to consider the influence of Shc on these reactions so far.
115	erbb11 · csrc → stat1	1		
116	erbb11 · csrc → stat3	1		
117	erbb11 · csrc → stat5	1		
118	erbb24 · csrc → stat5	1		
119	stat1 →	1		
120	stat3 →	1		
121	stat5 →	1		
**PI3K signaling**				
122	→ pi3kr	1	1	This reservoir represents the inactive form of PI3K that participates in the activation of Sos1_Eps8_E3b1 (see reaction 111).
123	erbb13 · pi3kr → pi3k	1		In Oda *et al* (2005) (second figure) activation of PI3K through all ErbB3- and ErbB4 dimers is considered. Indeed there are binding sites for the p85 subunit of PI3K on both ErbB3 and ErbB4 – however, there are 6 sites on ErbB3 and only one site on ErbB4, suggesting that ErbB3 is the main activator of PI3K (Olayioye *et al*, 2000). Furthermore, there are naturally occurring ErbB4 isoforms that do not contain the binding site for PI3K (Elenius *et al*, 1999) – thus we decided to include PI3K interaction only with ErbB3 dimers.
124	erbb23 · pi3kr → pi3k	1		
125	erbb34 · pi3kr → pi3k	1		
126	ras · pi3kr → pi3k	1		GTP-bound Ras activates PI3K (Downward, 1998b).
127	pi3kr · gab1 → pi3k	1		Phosphorylated Gab1 recruits and activates PI3K (Montagner *et al*, 2005). Gab1-bound SHP2 dephosphorylates the PI3K-binding site of Gab1. However, we decided not to include the negative influence of SHP2 on PI3K in the model so far, because it seems as if SHP2 indeed downregulates PI3K, but does not completely inhibit PI3K activation through Gab1 (Zhang *et al*, 2002; Montagner *et al*, 2005).

128	→ pten	1	1	PTEN and SHIP2 both down regulate PIP3 synthesis. As we could not find any information how PTEN and SHIP2 are regulated, we included them as inputs to the model. We suppose that down regulation of PI3K signaling is time delayed and therefore set the activation of PTEN and SHIP2 to time scale 2.
129	pten → ptend	2		
130	→ ship2	1	1	
131	ship2 → ship2d	2		
132	ship2d · !ptend · pi3k → pi34p2	1		PI3K phosphorylates PI(4,5)P2 at the D3 position and thus generates PIP3 (Vanhaesebroeck *et al*, 1997). Since PI(4,5)P2 is one of the major phosphorylated forms of PtdIns (Tolias & Cantley, 1999) we assume that it is always present in the cell and do not consider its regulation. PTEN dephosphorylates PIP3 at the D3 position (generating PI(4,5)P2), SHIP catalyzes the dephosphorylation at D5 (Scheid & Woodgett, 2003) and thus the synthesis of PI(3,4)P2. We assume that PTEN and SHIP2 do not compete for dephosphorylating PIP3, otherwise we would have to know which of the two reactions is preferred.
133	pi3k · !ptend · !ship2d → pip3	1		
134	→ pdk1	1	1	PDK1 appears to be constitutively active (Newton, 2003) and is thus an input to the model.
135	→ pp2a	1	0	
136	→ mtorr	1	1	mTOR can complex with rictor (see reactions 138 and 139) or raptor (see reaction 142) and therefore a reservoir of mTOR is included in the model.
137	mtorr → mtor_ric	1		As the molecular mechanism of the regulation of the mTOR-rictor complex is unknown (Sarbassov *et al*, 2005a), we assume that it is only activated by its reservoir and therefore always active (comparable to an external input to the model).
138	pdk1 · pip3 · !pp2a · mtor_ric → akt	1		PIP3 or PI(3,4)P2 recruit Akt and PDK1 to the plasma membrane. At the membrane, the HM region of Akt is phosphorylated at S473, probably by the Rictor-mTOR complex (Sarbassov *et al*, 2005b). The phosphorylated HM region of PKB stabilizes PDK1 so that PDK1 can phosphorylate T308 of PKB (Scheid & Woodgett, 2003). PP2A dephosphorylates Akt (Andjelković *et al*, 1996). Note that reaction 139 is not part of the map of Oda *et al* (2005).
139	pdk1 · pi34p2 · !pp2a · mtor_ric → akt	1		
140	!akt → tsc1_tsc2	1		Akt phosphorylates Tsc2 and thus inhibits the Rheb-GAP activity of the Tsc1/Tsc2 complex. GTP-bound Rheb activates the mTOR-raptor complex (Hay and Sonenberg, 2004).
141	!tsc1_tsc2 → rheb	1		
142	rheb · mtorr → mtor_rap	1		

143	erk12 → p70s6_1	1		Phosphorylation of several S/T residues (S404, S411, S418, S424, T421) in the C-terminal autoinhibitory domain of p70s6 leads to a conformational change that enables the phosphorylation of the catalytic sites T389 and S229 (Berven & Crouch, 2000). Both JNK and ERK1/2 are able to phosphorylate the autoinhibitory sites (Mukhopadhyay *et al*, 1992) – however, the mechanism of activation of these sites is not well understood and additional kinases are probably involved in this step (Berven & Crouch, 2000). We refer to p70s6 phosphorylated at the autoinhibitory sites as p70s6_1.<br>mTOR phosphorylates p70s6 on T389 (Hou *et al*, 2007) and T229 is phosphorylated by PDK1 (Downward, 1998a; Berven & Crouch, 2000).
144	jnk → p70s6_1	1		
145	pdk1 · mtor_rap · p70s6_1 → p70s6_2	1		
146	p70s6_2 →	1		
147	!pak1 · !akt→ bad	1		PAK1 phosphorylates Bad on S112 and S136, independently of PI3K (Schürmann *et al*, 2000). Akt phosphorylates Bad on S136. In some cell types (e. g. cerebellar granule cells) this suffices for inhibiting apoptosis. However, in other cell types (e. g. Il-3 dependent hematopoietic cells) Bad must be phosphorylated on S136 and S112 (Datta *et al*, 1997).
148	bad → pro_apoptotic	1		Phosphorylation of Bad avoids its proapoptotic function (Schürmann *et al*, 2000).
149	pro_apoptotic →	1		
**Activation of PKC**				
150	erbb11 → plcg	1		PLCγ is phosphorylated by ErbB1 at Y1254, Y783, Y771 and Y472 (Kim *et al*, 1990).
151	plcg → dag	1		PLCγ hydrolyzes PI(4,5)P2 to generate DAG and IP3 (Kim *et al*, 2000). At present, we do not consider PLCβ (as depicted in Oda *et al* (2005)), since this is part of the G-coupled receptor signaling.
152	plcg → ip3	1		
153	ip3 → ca	1		Binding of IP_3 to its receptor at the endoplasmatic reticulum leads to Ca^{2+} release into the cytosol (Alberts *et al*, 2004; Kim *et al*, 2000).
154	pdk1 · dag · ca → pkc	1		PKC is phosphorylated at its activation loop by PDK1. This leads to autophosphorylation and the release of PKC into the cytoplasma. A pseudosubstrate is bound to the substrate-binding cavity, which is released after binding of the second messengers Ca^{2+} and DAG (Newton, 2003). Note that the influence of calcium ions on this reaction is not part of the map of Oda *et al* (2005).
155	pkc →	1		

**MAPK**				
156	akt → aktd	2		Reaction that is only included for modeling the time delay Akt deactivates Raf1 with (see reactions 157 and 158).
157	ras · csrc · !aktd → raf1	1		Ras recruits Raf1 to the plasma membrane where it is phosphorylated at various sites. Src phosphorylates Raf1 on Y341, Pak1 phosphorylates S338, whereas it seems as if phosphorylation of either S338 or Y341 is sufficient for Raf1 activation. However, both kinases lead to different activation levels of Raf1 (the highest to be achieved in combination) that might stimulate different biological outcomes (King *et al*, 2001).
158	ras · pak1 · !aktd → raf1	1		
159	raccdc42 → mekk1	1		MEKK1 binds GTP-bound Cdc42/Rac and is thus activated (Schlesinger *et al*, 1998). Activated MEKK1 is autoubiquitinated, which blocks binding of downstream targets (Witowsky & Johnson, 2003). That is why we should think about setting this activation to 0 after some time.
160	raccdc42 → mekk4	1		MEKK4 contains a Cdc42/Rac interactive binding motif and is activated after binding to Cdc42/Rac. This binding is independent of the nucleotide bound to Cdc42/Rac, thus MEKK4 can be activated by the GDP-and GTP-bound protein (Schlesinger *et al*, 1998). Perhaps a different activation for Cdc42/Rac is necessary for binding MEKK4. We included only binding to the GTP-bound state in the model so far.
161	raccdc42 → mlk3	1		MLK3 binds GTP-bound Cdc42/Rac and is thus activated (Vacratsis *et al*, 2002).
162	mekk1 → mek12	1		MEKK1 and Raf1 both phosphorylate MEK1 and MEK2 (Schlesinger *et al*, 1998; Chen *et al*, 2001).
163	raf1 → mek12	1		
164	mek12 → erk12	1		MEK1 and MEK2 phosphorylate ERK1/2 (Robinson & Cobb, 1997).
165	mekk1 → mkk7	1		MEKK1 phosphorylates and thus activates MKK7 (Lu *et al*, 1997).
166	mekk1 → mkk4	1		MKK4 is phosphorylated by MLK3, MEKK1 (Tibbles *et al*, 1996) and MEKK4 (Gerwins *et al*, 1997).
167	mekk4 → mkk4	1		
168	mlk3 → mkk4	1		
169	mkk7 · mkk4→ jnk	1		MKK4 and MKK7 cooperate to activate JNK. MKK4 phosphorylates Y185, MKK7 phosphorylates T183 (Kishimoto *et al*, 2003).
170	mlk3 → mkk3	1		MLK3 phosphorylates and thus activates MKK3 and MKK6 (Tibbles *et al*, 1996).
171	mlk3 → mkk6	1		
172	mkk3 → p38	1		MKK3, MKK4 and MKK6 phosphorylate p38 on threonine and tyrosine residues (Raingeaud *et al*, 1996).
173	mkk4 → p38	1		
174	mkk6 → p38	1		
**Activation downstream of MAPK (mainly transcription factors)**				
175	→ mkp	1	0	
176	erk12 · !mkp → nucerk12	1		MKP dephosphorylates ERK1/2 and thus inhibits phosphorylation of transcription factors like Elk1 in the nucleus (Sun *et al*, 1993).
177	→ pp2b	1	0	Although PP2B is activated by Ca^{2+} (which is part of the model), we decided not to consider its regulation, because activation of PP2B also depends on calmodulin (Ishida *et al*, 2003).

178	nucerk12 · !pp2b → elk1	1		ERK1/2 phosphorylates Elk1 at S383 and S389 (Cavigelli *et al*, 1995). PP2B dephosphorylates Elk1 (Tian & Karin, 1999). In (Tian & Karin, 1999) activation of Elk1 through other MAPKs is also mentioned. However, for phosphorylating transcription factors translocation to the nucleus is necessary, which is stimulated in the case of JNK by UV-irradiation (Cavigelli *et al*, 1995).
179	elk1 →	1		
180	p38 → mk2	1		MK2 binds to and is phosphorylated by p38 (Gaestel, 2006).
181	mk2 → hsp27	1		MK2 phosphorylates Hsp27 on S15, S78 and S82 (Stokoe *et al*, 1992).
182	hsp27 →	1		
183	erk12 · pdk1 → p90rsk	1		ERK1/2 and PDK1 activate p90RSK by phosphorylation. ERK1/2 activates the C-terminal domain, PDK1 the N-terminal domain (Ser227), whereas the first is necessary for the latter (Froedin *et al*, 2000).
184	p90rsk → creb	1		p90RSK phosphorylates CREB on S133 and thus activates it (Cesare *et al*, 1998).
185	mk2 → creb	1		MK2 activates CREB through phosphorylation of S133 (Tan *et al*, 1996).
186	creb →	1		
187	!p90rsk · !akt → gsk3	1		p90Rsk phosphorylates Gsk3 on S9 and thus deactivates it in response to EGF. Akt also phosphorylates S9 - however, we are not sure whether this occurs only in response to insulin. Other kinases, like p70S6 and PKC, are also known to deactivate Gsk3; however, not in all cell types and not in response to Egf, so their influence has to be further studied before including it in the model (Grimes & Jope, 2001).
188	nucerk12 · !gsk3 → cmyc	1		ERK1/2 phosphorylates c-Myc at S62 and thus stabilizes it. The phosphorylation of S62 is necessary for the phosphorylation of T58 by GSK3 beta, which leads to ubiquitin dependent degradation of c-Myc (Sears *et al*, 2000). GSK3 beta is not included in Oda *et al* (2005).
189	cmyc →	1		
190	jnk → cjun	1		JNK phosphorylates c-Jun. Unphosphorylated c-Jun is ubiquitinated and degraded. Phosphorylation by JNK also increases the transriptional activity of c-Jun (Karin *et al*, 1997). Regulation of transcription of c-Jun is not considered here, this could be included in a model regarding multi-level activation.
191	!pp2a · jnk → cfos	1		JNK phosphorylates c-Fos and thus prevents it from degradation (Coronella-Wood *et al*, 2004). Note that this reaction is independent of ERK (Coronella-Wood *et al*, 2004). PP2A dephosphorylates c-Fos, whereas PP2B does not (Coronella-Wood *et al*, 2004) inconsistent with Oda *et al* (2005). Regulation of transcription of c-Fos is not considered here, this could be included in a model regarding multi-level activation.

192	!pp2a · p90rsk · erk12 → cfos	1		ERK1/2 and p90RSK coordinately phosphorylate c-Fos - unphosphorylated c-Fos is rapidly degraded (Murphy *et al*, 2002). Note that the influence of p90RSK is not depicted in Oda *et al* (2005). PP2A dephosphorylates cfos, whereas PP2B does not (Coronella-Wood *et al*, 2004) – inconsistent with Oda *et al* (2005). Regulation of transcription of c-Fos is not considered here, this could be included in a model regarding multi-level activation.
193	cfos · cjun → ap1	1		c-Jun and c-Fos heterodimerize and form the transcription factor AP-1 (Karin *et al*, 1997).
194	ap1 →	1		
**Endocytosis**				
195	erbb11 → ccbl	1		c-Cbl binds ErbB1 at pY1045, leading to degradation of the receptor in the lysosome (Citri & Yarden, 2006).
196	eps8r · erbb11 → rntre	1		RN-tre binds to the adaptor protein Eps8 and is phosphorylated in response to EGF stimulation (Lanzetti *et al*, 2000).
197	ras → rin1	1		Rab5-GEF activity of Rin1 is potentiated by activated Ras (Tall *et al*, 2001).
198	!rntre · rin1 → rab5a	1		Rin1 mediates GDP/GTP exchange for Rab5a thus activating it (Tall *et al*, 2001). The GTPase activating protein RN-tre acts on Rab5a and inhibits internalization of the EGFR (Lanzetti *et al*, 2000).
199	ccbl · rab5a → endocyt_degrad	1		c-Cbl and Rab5a are both involved in the endocytic trafficking of ErbB receptors. c-Cbl is necessary for degradation of the receptors, while Rab5a controls the formation and fusion of endocytic vesicles (Citri & Yarden, 2006).
**Actin reorganization**				
200	grb2 · raccdc42 → pak1	1		Pak1 is recruited to the plasma membrane via Grb2 (Puto *et al*, 2003) or Nck (Li *et al*, 2001) where it is activated through GTP-bound Rac/Cdc42 (Edwards *et al*, 1999). Note that reaction 201 is not included in Oda *et al* (2005).
201	nck · raccdc42 → pak1	1		
202	pak1 → limk1	1		PAK1 (activated through Rac/Cdc42) phosphorylates LIMK1 at T508 (Edwards *et al*, 1999).
203	limk1 → actinreorg	1		LIMK1 phosphorylates cofilin, thereby leading to accumulation of actin filaments and aggregates (Edwards *et al*, 1999).
204	actinreorg →	1		

Supplementary References

Agazie YM, Hayman MJ (2003). Molecular mechanism for a role of shp2 in epidermal growth factor receptor signaling. *Mol Cell Biol* **23**: 7875-7886

Alberts B, Johnson A, Lewis J, Raff M, Roberts K, Walter P (2004). *Molekularbiologie der Zelle.* Wiley VCH Weinheim

Alroy I, Yarden Y (1997). The erbb signaling network in embryogenesis and oncogenesis: signal diversification through combinatorial ligand-receptor interactions. *FEBS Lett* **410**: 83–86

Andjelković M, Jakubowicz T, Cron P, Ming XF, Han JW, Hemmings BA (1996) Activation and phosphorylation of a pleckstrin homology domain containing protein kinase (RAC-PK/PKB) promoted by serum and protein phosphatase inhibitors. *Proc Natl Acad Sci U S A* **93**: 5699-5704

Beerli RR, Hynes NE (1996). Epidermal growth factor-related peptides activate distinct subsets of erbb receptors and differ in their biological activities. *J Biol Chem* **271**: 6071-6076

Berven LA, Crouch MF (2000). Cellular function of p70s6: a role in regulating cell motility. *Immunol Cell Biol* **78**: 447-451

Cavigelli M, Dolfi F, Claret FX, Karin M (1995). Induction of c-fos expression through jnk-mediated tcf/elk-1 phosphorylation. *EMBO J* **14**: 5957–5964

De Cesare D, Jacquot S, Hanauer A, Sassone-Corsi P (1998). Rsk-2 activity is necessary for epidermal growth factor-induced phosphorylation of creb protein and transcription of c-fos gene. *Proc Natl Acad Sci U S A* **95**: 12202–12207

Chen Z, Gibson TB, Robinson F, Silvestro L, Pearson G, Xu B, Wright A, Vanderbilt C, Cobb M (2001). MAP Kinases. *Chem Rev* **101**: 2449-2476

Citri A, Yarden Y (2006). Egf-erbb signalling: towards the systems level. *Nat Rev Mol Cell Biol* **7**: 505–516

Coronella-Wood J, Terrand J, Sun H, Chen QM (2004). c-fos phosphorylation induced by h2o2 prevents proteasomal degradation of c-fos in cardiomyocytes. *J Biol Chem* **279**: 33567–33574

Cox AD, Der CJ (2003). The dark side of ras: regulation of apoptosis. *Oncogene* **22**: 8999–9006

Datta SR, Dudek H, Tao X, Masters S, Fu H, Gotoh Y, Greenberg ME (1997). Akt phosphorylation of bad couples survival signals to the cell-intrinsic death machinery. *Cell* **91**: 231–241

Douville E, Downward J (1997). Egf induced sos phosphorylation in pc12 cells involves p90 rsk-2. *Oncogene* **15**: 373–383

Downward J (1998). Lipid-regulated kinases: some common themes at last. *Science* **279**: 673– 674

Downward J (1998). Ras signalling and apoptosis. *Curr Opin Genet Dev* **8**: 49–54

Edwards DC, Sanders LC, Bokoch GM, Gill GN (1999). Activation of lim-kinase by pak1 couples rac/cdc42 gtpase signalling to actin cytoskeletal dynamics. *Nat Cell Biol* **1**: 253–259

Elenius K, Choi CJ, Paul S, Santiestevan E, Nishi E, Klagsbrun M (1999). Characterization of a naturally occuring erbb4 isoform that does not bind or activate phosphatidyl inositol 3-kinase. *Oncogene* **18**: 2607-2615

Froedin M, Jensen CJ, Merienne K, Gammeltoft S (2000). A phosphoserine-regulated docking site in the protein kinase rsk2 that recruits and activates pdk1. *EMBO J* **19**: 2924–2934

Gaestel M (2006). Mapkap kinases – MKs – two's company, three's a crowd. *Nat Rev Mol Cell Biol* **7**: 120-130

Gerwins P, Blank JL, Johnson GL (1997). Cloning of a novel mitogen-activated protein kinase kinase kinase, mekk4, that selectively regulates the c-jun amino terminal kinase pathway. *J Biol Chem* **272**: 8288–8295

Graus-Porta D, Beerli RR, Daly JM, Hynes NE (1997). Erbb-2, the preferred heterodimerization partner of all erbb receptors, is a mediator of lateral signaling. *EMBO J* **16**: 1647–1655

Grimes CA, Jope RS (2001). The multifaceted roles of glycogen synthase kinase 3beta in cellular signaling. *Prog Neurobiol* **65**: 391–426

Harari D, Tzahar E, Romano J, Shelly M, Pierce JH, Andrews GC, Yarden Y (1999). Neuregulin-4: a novel growth factor that acts through the erbb-4 receptor tyrosine kinase. *Oncogene* **18**: 2681–2689

Hay N, Sonenberg N (2004). Upstream and downstream of mTOR. *Genes Dev* **18**: 1926-1945

Hou Z, He L, Qi RZ (2007). Regulation of s6 kinase 1 activation by phosphorylation at ser-411. *J Biol Chem* **282**: 6922-6928

Innocenti M, Frittoli E, Ponzanelli I, Falck JR, Brachmann SM, Di Fiore PP, Scita G (2003). Phosphoinositide 3-kinase activates rac by entering in a complex with eps8, abi1, and sos-1. *J Cell Biol* **160**: 17–23

Ishida A, Shigeri Y, Taniguchi T, Kameshita I (2003). Protein phosphatases that regulate multifunctional ca2+/calmodulin-dependent protein kinases: from biochemistry to pharmacology. *Pharmacol Ther* **100**: 291–305

Jones JT, Akita RW, Sliwkowski MX (1999). Binding specificities and affinities of egf domains for erbb receptors. *FEBS Lett* **447**: 227–231

Karin M, Liu Z, Zandi E (1997). Ap-1 function and regulation. *Curr Opin Cell Biol* **9**: 240–246

Keilhack H, Tenev T, Nyakatura E, Godovac-Zimmermann J, Nielsen L, Seedorf K, Böhmer FD (1998). Phosphotyrosine 1173 mediates binding of the protein-tyrosine phosphatase shp-1 to the epidermal growth factor receptor and attenuation of receptor signaling. *J Biol Chem* **273**: 24839–24846

Kim MJ, Kim E, Ryu SH, Suh PG (2000). The mechanism of phospholipase C-gamma 1 regulation. *Exp Mol Med* **32:** 101-109

Kim JW, Sim SS, Kim UH, Nishibe S, Wahl MI, Carpenter G , Rhee SG (1990). Tyrosine residues in bovine phospholipase c-gamma phosphorylated by the epidermal growth factor receptor in vitro. *J Biol Chem* **265:** 3940–3943

King AJ, Wireman RS, Hamilton M, Marshall MS (2001). Phosphorylation site specificity of the pak-mediated regulation of raf-1 and cooperativity with src. *FEBS Lett* **497**: 6–14

Kishimoto H, Nakagawa K, Watanabe T, Kitagawa D, Momose H, Seo J, Nishitai G, Shimizu N, Ohata S, Tanemura S, Asaka S, Goto T, Fukushi H, Yoshida H, Suzuki A, Sasaki T, Wada T, Penninger JM, Nishina H, Katada T (2003) Different properties of SEK1 and MKK7 in dual phosphorylation of stress-induced activated protein kinase SAPK/JNK in embryonic stem cells. *J Biol Chem* **278**: 16595-16601

Landau M, Ben-Tal N (2008) Dynamic equilibrium between multiple active and inactive conformations explains regulation and oncogenic mutations in ErbB receptors. *Biochim Biophys Acta* **1785**: 12-31

Lanzetti L, Rybin V, Malabarba MG, Christoforidis S, Scita G, Zerial M, Di Fiore PP (2000). The eps8 protein coordinates egf receptor signalling through rac and trafficking through rab5. *Nature* **408**: 374–377

Lenferink E, Pinkas-Kramarski R, van de Poll ML, van Vugt MJ, Klapper LN, Tzahar E, Waterman H, Sela M, van Zoelen EJ, Yarden Y (1998). Differential endocytic routing of homo-and hetero-dimeric erbb tyrosine kinases confers signaling superiority to receptor heterodimers. *EMBO J* **17**: 3385–3397

Li N, Batzer A, Daly R, Yajnik V, Skolnik E, Chardin P, Bar-Sagi D, Margolis B, Schlessinger J (1993). Guanine-nucleotide-releasing factor hsos1 binds to grb2 and links receptor tyrosine kinases to ras signalling. *Nature* **363**: 85–88

Li W, Fan J, Woodley DT (2001). Nck/Dock: an adapter between cell surface receptors and the actin cytoskeleton. *Oncogene* **20**: 6403-6417

Lu X, Nemoto S, Lin A (1997). Identification of c-jun nh2-terminal protein kinase (jnk)-activating kinase 2 as an activator of jnk but not p38. *J Biol Chem* **272**: 24751–24754

Montagner A, Yart A, Dance M, Perret B, Salles JP, Raynal P (2005). A novel role for gab1 and shp2 in epidermal growth factor-induced ras activation. *J Biol Chem* **280**: 5350–5360

Mukhopadhyay NK, Price DJ, Kyriakis JM, Pelech S, Sanghera J, Avruch J (1992). An array of insulin-activated, proline-directed serine/threonine protein kinases phosphorylate the p70 s6 kinase. *J Biol Chem* **267**: 3325-3335

Murphy LO, Smith S, Chen RH, Fingar DC, Blenis J (2002). Molecular interpretation of erk signal duration by immediate early gene products. *Nat Cell Biol* **4**: 556–564

Newton AC (2003). Regulation of the abc kinases by phosphorylation: protein kinase c as a paradigm. *Biochem J* **370**: 361–371

Oda K, Matsuoka Y, Funahashi A, and Hiroaki Kitano H (2005). A comprehensive pathway map of epidermal growth factor receptor signaling. *Mol Syst Biol* **1:** 2005.0010

Okabayashi Y, Kido Y, Okutani T, Sugimoto Y, Sakaguchi K, Kasuga M (1994). Tyrosines 1148 and 1173 of activated human epidermal growth factor receptors are binding sites of shc in intact cells. *J Biol Chem* **269**: 18674–18678

Olayioye MA, Beuvink I, Horsch K, Daly JM, Hynes NE (1999). Erbb receptor-induced activation of stat transcription factors is mediated by src tyrosine kinases. *J Biol Chem* **274**: 17209–17218

Olayioye MA, Graus-Porta D, Beerli RR, Rohrer J, Gay B, Hynes NE (1998). Erbb-1 and erbb-2 acquire distinct signaling properties dependent upon their dimerization partner. *Mol Cell Biol* **18**: 5042–5051

Olayioye MA, Neve RM, Lane HA, Hynes NE (2000). The erbb signaling network: receptor heterodimerization in development and cancer. *EMBO J* **19**: 3159–3167

Pinkas-Kramarski R, Shelly M, Guarino BC, Wang LM, Lyass L, Alroy I, Alimandi M, Kuo A, Moyer JD, Lavi S, Eisenstein M, Ratzkin BJ, Seger R, Bacus SS, Pierce JH, Andrews GC, Yarden Y, Alamandi M (1998). Erbb tyrosine kinases and the two neuregulin families constitute a ligand-receptor network. *Mol Cell Biol* **18**: 6090–6101

Puto LA, Pestonjamasp K, King CC, Bokoch GM (2003). p21-activated kinase 1 (pak1) interacts with the grb2 adapter protein to couple to growth factor signaling. *J Biol Chem* **278**: 9388–9393

Raingeaud J, Whitmarsh AJ, Barrett T, Drijard B, Davis RJ (1996). Mkk3-and mkk6 regulated gene expression is mediated by the p38 mitogen-activated protein kinase signal transduction pathway. *Mol Cell Biol* **16**: 1247–1255

Robinson MJ, Cobb MH (1997). Mitogen-activated protein kinase pathways. *Curr Opin Cell Biol* **9**: 180–186

Rodrigues GA, Falasca M, Zhang Z, Ong SH, Schlessinger J (2000). A novel positive feedback loop mediated by the docking protein gab1 and phosphatidylinositol 3-kinase in epidermal growth factor receptor signaling. *Mol Cell Biol* **20**: 1448–1459

Saez-Rodriguez J, Goldsipe A, Muhlich J, Alexopoulos L, Millard B, Lauffenburger DA, Sorger PK (2008). Flexible Informatics for Linking Experimental Data to Mathematical Models via DataRail. *Bioinformatics* **24:** 840-847

Sarbassov DD, Ali SM, Sabatini DM (2005a). Growing roles for the mTOR pathway. *Curr Opin Cell Biol* **17**: 596-603.

Sarbassov DD, Guertin DA, Ali SM, Sabatini DM (2005b). Phosphorylation and regulation of Akt/PKB by the rictor-mTOR complex. *Science* **307**: 1098-1101.

Sato K, Nagao T, Kakumoto M, Kimoto M, Otsuki T, Iwasaki T, Tokmakov AA, Owada K, Fukami Y (2002). Adaptor protein shc is an isoform-specific direct activator of the tyrosine kinase c-src. *J Biol Chem* **277**: 29568– 29576

Sears R, Nuckolls F, Haura E, Taya Y, Tamai K, Nevins JR (2000). Multiple ras-dependent phosphorylation pathways regulate myc protein stability. *Genes Dev* **14**: 2501-2514

Scheid MP, Woodgett JR (2003). Unravelling the activation mechanisms of protein kinase b/akt. *FEBS Lett* **546**: 108–112

Schlesinger TK, Fanger GR, Yujiri T, Johnson GL (1998). The tao of mekk. *Front Biosci*, **3**: D1181–D1186

Schürmann A, Mooney AF, Sanders LC, Sells MA, Wang HG, Reed JC, Bokoch GM (2000). p21-activated kinase 1 phosphorylates the death agonist bad and protects cells from apoptosis. *Mol Cell Biol* **20**: 453–461

Schulze WX, Deng L, Mann M (2005) Phosphotyrosine interactome of the ErbB-receptor kinase family. *Mol Syst Biol* **1:** 2005.0008.

Shelly M, Pinkas-Kramarski R, Guarino BC, Waterman H, Wang LM, Lyass L, Alimandi M, Kuo A, Bacus SS, Pierce JH, Andrews GC, Yarden Y (1998). Epiregulin is a potent pan-erbb ligand that preferentially activates heterodimeric receptor complexes. *J Biol Chem* **273**: 10496–10505

Stokoe D, Engel K, Campbell DG, Cohen P, Gaestel M (1992). Identification of mapkap kinase 2 as a major enzyme responsible for the phosphorylation of the small mammalian heat shock proteins. *FEBS Lett* **313**: 307-313

Sun H, Charles CH, Lau LF, Tonks NK (1993). Mkp-1 (3ch134), an immediate early gene product, is a dual specificity phosphatase that dephosphorylates map kinase in vivo. *Cell* **75**: 487–493

Tall GG, Barbieri MA, Stahl PD, Horazdovsky BF (2001). Ras-activated endocytosis is mediated by the rab5 guanine nucleotide exchange activity of rin1. *Dev Cell* **1**: 73–82

Tamás P, Solti Z, Bauer P, Illés A, Sipeki S, Bauer A, Faragó A, Downward J, Buday L (2003). Mechanism of epidermal growth factor regulation of vav2, a guanine nucleotide exchange factor for rac. *J Biol Chem* **278**: 5163–5171

Tan Y, Rouse J, Zhang A, Cariati S, Cohen P, Comb MJ (1996). Fgf and stress regulate creb and atf-1 via a pathway involving p38 map kinase and mapkap kinase-2. *EMBO J* **15**: 4629-4642

Tian J, Karin M (1999). Stimulation of elk1 transcriptional activity by mitogen-activated protein kinases is negatively regulated by protein phosphatase 2b (calcineurin). *J Biol Chem* **274**: 15173–15180

Tibbles LA, Ing YL, Kiefer F, Chan J, Iscove N, Woodgett JR, Lassam NJ (1996). Mlk3 activates the sapk/jnk and p38/rk pathways via sek1 and mkk3/6. *EMBO J* **15**: 7026– 7035

Tolias KF, Cantley LC (1999). Pathways for phosphoinositide synthesis. *Chem Phys Lipids* **98**: 69–77

Vacratsis PO, Phinney BS, Gage DA, Gallo KA (2002). Identification of in vivo phosphorylation sites of mlk3 by mass spectrometry and phosphopeptide mapping. *Biochemistry* **41**: 5613–5624

Vanhaesebroeck B, Leevers SJ, Panayotou G, Waterfield MD (1997). Phosphoinositide 3-kinases: a conserved family of signal transducers. *Trends Biochem Sci* **22**: 267–272

Wang LM, Kuo A, Alimandi M, Veri MC, Lee CC, Kapoor V, Ellmore N, Chen XH, Pierce JH (1998). Erbb2 expression increases the spectrum and potency of ligand-mediated signal transduction through erbb4. *Proc Natl Acad Sci U S A* **95**: 6809–6814

Witowsky JA, Johnson GL (2003). Ubiquitylation of mekk1 inhibits its phosphorylation of mkk1 and mkk4 and activation of the erk1/2 and jnk pathways. *J Biol Chem* **278**: 1403– 1406

Zhang SQ, Tsiaras WG, Araki T, Wen G, Minichiello L, Klein R, Neel BG (2002). Receptor-specific regulation of phosphatidylinositol 3’-kinase activation by the protein tyrosine phosphatase shp2. *Mol Cell Biol* **22**: 4062– 4072
