# Supplementary material for: The Logic of EGFR/ErbB Signaling: Theoretical Properties and Analysis of High-Throughput Data
Source: PLoS Comput Biol. 2009 Aug 7;5(8):e1000438. doi: 10.1371/journal.pcbi.1000438 (PMC2710522; doi:10.1371/journal.pcbi.1000438)
Supplement: Table S2 — Incomplete truth tables (ITTs) in the model variant M2. (0.01 MB PDF) [file pcbi.1000438.s006.pdf]

Table S2. Incomplete truth tables (ITTs) in model M2.

All gates from model M1 (see Table S1.2) that point into nodes given in column 1 (nodes) are replaced by the interactions given in column 2. ITT gates are denoted with *, AND gates with ·.

**Nodes**	**interactions**
endocyt_degrad	ccbl * rab5a → endocyt_degrad
RN-tre	erbb11 * eps8r → rntre
Rac/Cdc42	sos1_eps8_e3b1 * vav2 → rac_cdc42
Raf1	pak1 * csrc → pak1crscd
	!aktd · pak1csrcd · ras → raf1
p70s6 kinase (autoinhibitory domain)	jnk * erk → p70s6_1
CREB	p90rsk * mk2 → creb
c-Fos	jnk * erk * p90rsk → jnkerkp90rskd
	jnkerkp90rskd · !pp2a → cfos
Sos1	p90rsk * erk12 → p90rskerk12d
	!p90rskerk12d · grb2 · sos1r → sos1
MKK4	mekk1 * mekk4 * mlk3 → mkk4
p38	mkk4 * mkk3 * mkk6 → p38
Gsk3	!akt * !p90rsk → gsk3
BAD	!akt * !pak1 → bad
PKC	dag * ca → dagcad
	dagcad · pdk1 → pkc
MEK1/2	raf1 * mekk1 → mek12
