# Supplementary material for: The Logic of EGFR/ErbB Signaling: Theoretical Properties and Analysis of High-Throughput Data
Source: PLoS Comput Biol. 2009 Aug 7;5(8):e1000438. doi: 10.1371/journal.pcbi.1000438 (PMC2710522; doi:10.1371/journal.pcbi.1000438)
Supplement: Table S3 — Proposed model changes to improve the fit of the model to the data. (0.01 MB PDF) [file pcbi.1000438.s007.pdf]

Table S3. Proposed model changes to improve the fit of the model to the data.

3.1. Proposed model changes for primary hepatocytes.

Interaction in Table S1.2	Interaction	Changed to
108	erbb11 · pip3 → vav2	erbb11 → vav2
109	erbb11 · pi34p2 → vav2	
113	sos1eps8e3b1 → raccdc42	removed
116	erbb11 · csrc → stat3	removed
184	p90rsk → creb	tgfa → creb (unknown mechanism)
185	mk2 → creb	
187	!p90rsk · !akt → gsk3	!pkc → gsk3
**Additional interaction**		
mtor_rap → p70s6_1 (excluded in logical analysis; in addition to reactions 143/144 in Table S1.2)		

3.2. Proposed model changes for HepG2.

Interaction in Table S1.2	Interaction	Changed to
108	erbb11 · pip3 → vav2	erbb11 → vav2
109	erbb11 · pi34p2 → vav2	
113	sos1eps8e3b1 → raccdc42	removed
116	erbb11 · csrc → stat3	removed
181	mk2 → hsp27	removed
185	mk2 → creb	tgfa → creb (unknown mechanism)
187	!p90rsk · !akt → gsk3	!pkc → gsk3
**Additional interactions**		
pi3k → jnk (unknown mechanism)		
mtor_rap → p70s6_1 (excluded in logical analysis; in addition to reactions 143/144 in Table S1.2)		
